# Atomic cobalt on nitrogen-doped graphene for hydrogen generation

**DOI:** 10.1038/ncomms9668

**Published:** 2015-10-21

**Authors:** Huilong Fei, Juncai Dong, M. Josefina Arellano-Jiménez, Gonglan Ye, Nam Dong Kim, Errol L.G. Samuel, Zhiwei Peng, Zhuan Zhu, Fan Qin, Jiming Bao, Miguel Jose Yacaman, Pulickel M. Ajayan, Dongliang Chen, James M. Tour

**Affiliations:** 1 Department of Chemistry, Rice University, 6100 Main Street, Houston, Texas 77005, USA; 2 Beijing Synchrotron Radiation Facility, Institute of High Energy Physics, Chinese Academy of Sciences, Beijing 100049, China; 3 Department of Physics and Astronomy, University of Texas at San Antonio, One UTSA Circle, San Antonio, Texas 78249, USA; 4 Department of Materials Science and NanoEngineering, Rice University, 6100 Main Street, Houston, Texas 77005, USA; 5 Department of Electrical and Computer Engineering, University of Houston, Houston, Texas 77204, USA; 6 Smalley Institute for Nanoscale Science and Technology, Rice University, 6100 Main Street, Houston, Texas 77005, USA

## Abstract

Reduction of water to hydrogen through electrocatalysis holds great promise for clean energy, but its large-scale application relies on the development of inexpensive and efficient catalysts to replace precious platinum catalysts. Here we report an electrocatalyst for hydrogen generation based on very small amounts of cobalt dispersed as individual atoms on nitrogen-doped graphene. This catalyst is robust and highly active in aqueous media with very low overpotentials (30 mV). A variety of analytical techniques and electrochemical measurements suggest that the catalytically active sites are associated with the metal centres coordinated to nitrogen. This unusual atomic constitution of supported metals is suggestive of a new approach to preparing extremely efficient single-atom catalysts.

## Abstract (web summary)

There is ongoing interest in the development of non-precious metal catalysts for hydrogen evolution. Here, the authors fabricate a cobalt catalyst in which the cobalt is dispersed as single atoms on nitrogen-doped graphene, and report its high activity and stability for water reduction in acidic media.

## Graphical abstract

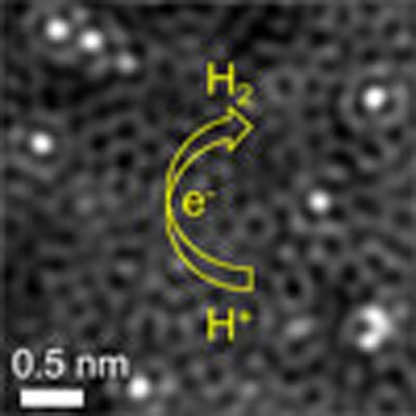

Electrochemical reduction of water through the hydrogen evolution reaction (HER) is a clean and sustainable approach to generate molecular hydrogen (H_2_), which has been proposed as a future energy carrier^1^,^2^. Catalysts are needed to improve HER efficiency by minimizing reaction kinetic barriers, which manifest themselves as overpotentials (*η*). Although platinum (Pt) is the most active HER catalyst, its scarcity and high cost limit its widespread use. Thus, the transition to a hydrogen economy calls for alternative electrocatalysts based on earth-abundant elements, such as non-precious metal oxides^3^,^4^, sulfides^5^, phosphides^6^,^7^, carbides^8^ and borides^9^. In spite of their low *η* for HER, the active sites of these inorganic-solid catalysts, like other heterogeneous catalysts, are sparsely distributed at selective sites (that is, surface sites or edges sites)^10^,^11^. To expose more active sites, these catalysts are generally downsized into nanoparticulate form and stabilized onto certain substrates^12^,^13^. Graphene is such a substrate that has a large specific surface area (high catalyst loading), good stability (tolerance to harsh operational conditions) as well as a high electrical conductivity (facilitated electron transfer) and therefore has been widely used to disperse nanoparticles for advanced electrocatalysis^14^,^15^,^16^.

The dispersing ability of graphene is, however, far from being fulfilled unless single-atom catalysis (SAC) is achieved. SAC represents the lowest size limit to obtain full atom utility in a catalyst and has recently emerged as a new research frontier^17^. Although an increasing number of SAC systems have been reported, most have been focusing on supporting noble metal atoms (for example, Pt, Au, Pd) on metal oxide or metal surfaces with a limited number of applications demonstrated^18^,^19^,^20^,^21^,^22^,^23^. Wide employment of SAC is hampered mainly due to the lack of readily available synthetic approaches originated from the aggregation tendency of single atoms. Here, we report an inexpensive, concise and scalable method to disperse the earth-abundant metal, cobalt, onto nitrogen-doped graphene (denoted as Co-NG) by simply heat-treating graphene oxide (GO) and small amounts of cobalt salts in a gaseous NH_3_ atmosphere. These small amounts of cobalt atoms, coordinated to nitrogen atoms on the graphene, can work as extraordinary catalysts towards HER in both acidic and basic water.

## Results

### Synthesis and characterization of the Co-NG catalyst

**Figure 1:**
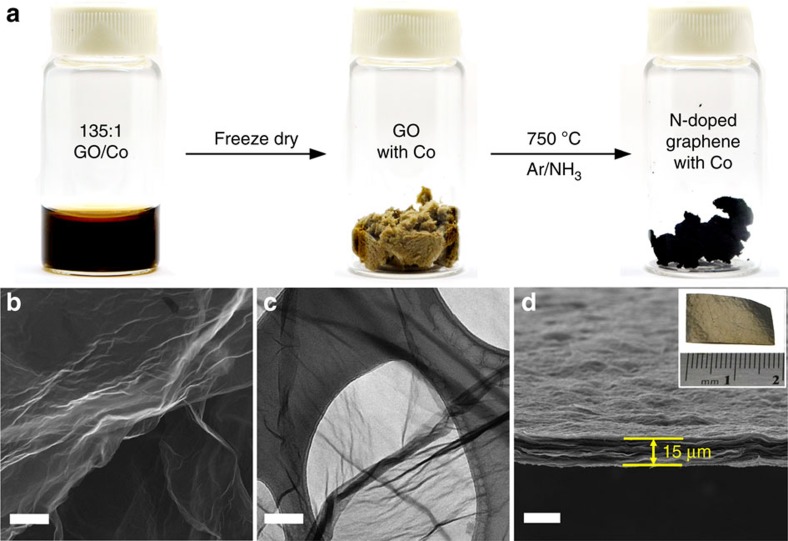
Preparation and morphology characterizations. (**a**) Schematic illustration of the synthetic procedure of the Co-NG catalyst. (**b**) SEM image of the Co-NG nanosheets. Scale bar, 2 μm. (**c**) TEM image of the Co-NG nanosheets atop a lacey carbon TEM grid. Scale bar, 50 nm. (**d**) SEM image showing the cross-section view of the Co-NG paper with thickness of 15 μm, prepared by filtration of Co-containing GO suspension followed by NH_3_ annealing. Scale bar, 20 μm. The inset shows the optical image of a 2 × 1 cm^2^ Co-NG paper.

To prepare the Co-NG catalyst, a precursor solution was first prepared by sonicating GO and cobalt salts (CoCl_2_·6H_2_O; weight ratio GO/Co=135:1) in water. The well-mixed precursor solution, as depicted in Fig. 1a, was then freeze-dried to minimize re-stacking of the GO sheets. The Co-NG catalyst was finally obtained by heating the dried sample under a NH_3_ atmosphere to dope the GO with nitrogen. Control samples of nitrogen-doped graphene (NG) and Co-containing graphene (Co-G, with no N doping) were also prepared. A detailed preparation procedure is described in the Methods section. The morphology of the Co-NG was examined by scanning electron microscopy (SEM); Fig. 1b reveals that the Co-NG has similar morphologic features to graphene with sheet-like structures. Transmission electron microscopy (TEM; Fig. 1c) shows Co-NG nanosheets with ripples observed on the surface. No cobalt-derived particles were found by SEM or TEM on the Co-NG nanosheets, underscoring the smallness in size of the Co. The Co-NG could be formed into a paper by filtration of Co-containing GO suspension and subsequent NH_3_ treatment (Fig. 1d).

**Figure 2:**
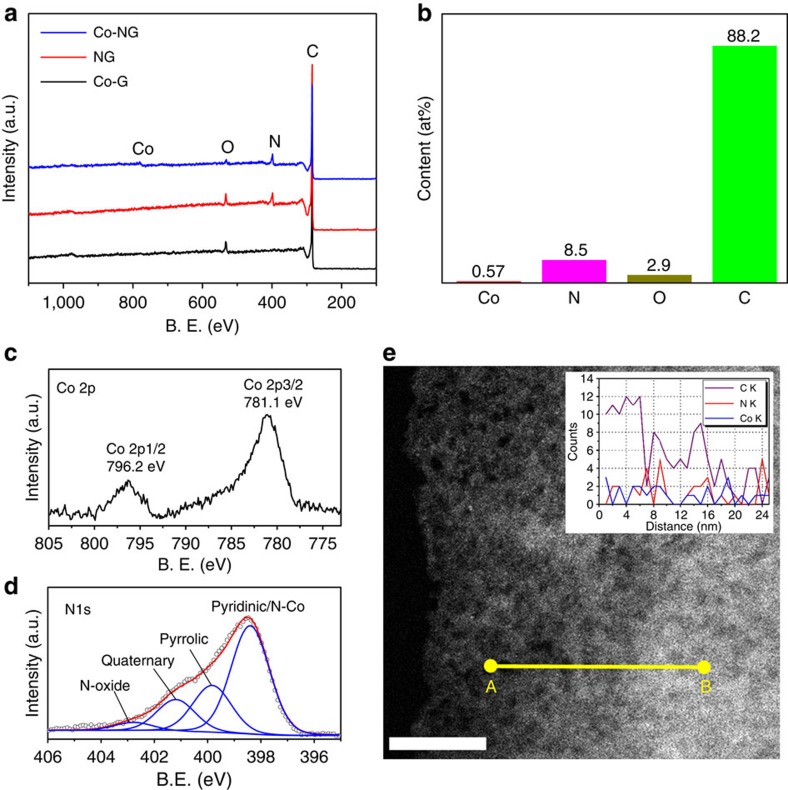
Compositional characterizations on the Co-NG. (**a**) XPS survey spectra of the Co-NG, NG and Co-G. (**b**) Chart showing the percentages of cobalt, nitrogen, oxygen and carbon in the Co-NG measured by XPS and ICP-OES. (**c**,**d**) High-resolution XPS Co 2p and N 1s spectra, respectively. (**e**) STEM image of the Co-NG nanosheet. Scale bar, 20 nm. Inset is the EDS elemental line scan from A to B showing the presence of C, N and Co elements.

To probe the compositions of Co-NG, X-ray photoelectron spectroscopy (XPS; Fig. 2a) showed the presence of C, N and O peaks in the samples of Co-NG and NG, whereas the N peak was absent in Co-G. No significant signals were found at the Co region in the Co-NG. To determine the Co content, inductively coupled plasma optical emission spectrometry (ICP-OES) was performed after digesting the powdered sample in HNO_3_. By combined use of XPS and ICP-OES, the Co-NG was determined to be 0.57 at% Co, 8.5 at% N, 2.9 at% O and 88.2 at% C, as summarized in Fig. 2b. The Co content in NG with no intentional addition of Co is negligible (<0.005 at% by ICP-OES). The XPS detailed scan in the Co region (Fig. 2c) of Co-NG shows two peaks at a binding energy of 781.1 and 796.2 eV, corresponding to the 2*p*_3/2_ and 2*p*_1/2_ levels, respectively. The peak positions and the separation of 15.1 eV between these two peaks indicates the presence of Co(III)^24^. The N 1s (Fig. 2d) can be deconvoluted into different types of nitrogen^25^,^26^, namely pyridinic and N-Co (398.4 eV), pyrrolic (399.8 eV), graphitic (401.2 eV) and N-oxide (402.8). The small difference in the binding energies between pyridinic N and N-Co prevents further deconvolution^27^. From the peak intensity, the N was dominated by the pyridinic/N-Co species. The C 1s and O 1s XPS were shown in Supplementary Fig. 1. The presence of Co and N was further confirmed by the energy-dispersive X-ray spectroscopy (EDS) spectrum (Supplementary Fig. 2) taken in the area shown in Fig. 2e of the scanning transmission electron microscopy (STEM) image. The EDS line scan in Fig. 2e reveals the close-proximity distributions of the Co and N elements.

### Atomic structure analysis by HAADF and EXAFS

**Figure 3:**
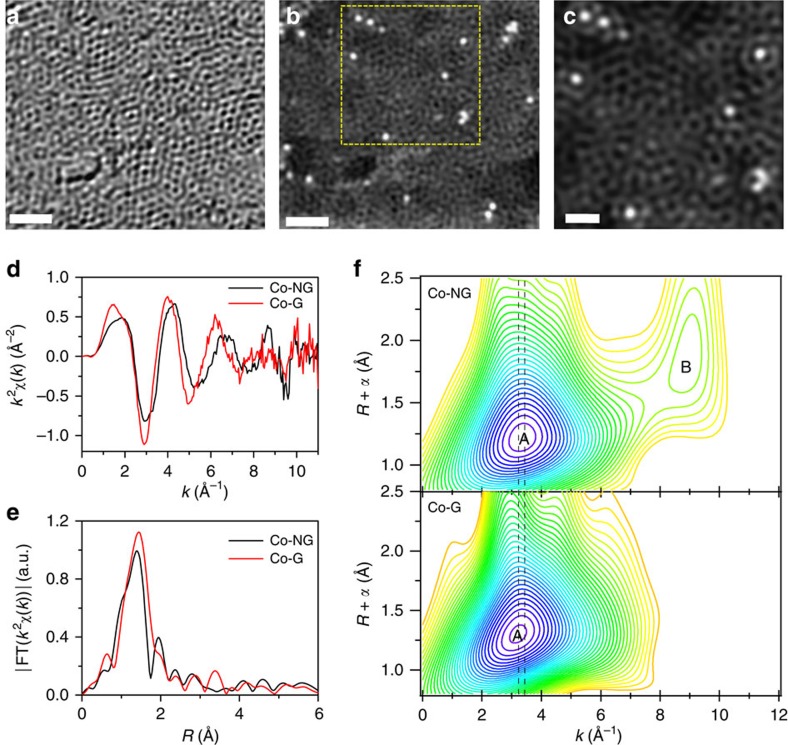
Structural characterizations on the Co-NG. (**a**) Bright-field aberration-corrected STEM image of the Co-NG showing the defective and disordered graphitic carbon structures. Scale bar, 1 nm. (**b**) HAADF-STEM image of the Co-NG, showing many Co atoms well-dispersed in the carbon matrix. Scale bar, 1 nm. (**c**) The enlarged view of the selected area in **b**. Scale bar, 0.5 nm. (**d**,**e**) The *k*^2^-weighted EXAFS in *k*-space and their Fourier transforms in *R* space for the Co-NG and Co-G, respectively. (**f**) Wavelet transforms for the Co-NG and Co-G. The location of the maximum A shifts from 3.2 Å^−1^ for Co-G to 3.4 Å^−1^ for Co-NG, indicating the presence of Co-N bonding in Co-NG. The vertical dashed lines are provided to guide the eye.

To investigate the atomic structure of the Co-NG nanosheet, we used high-angle annular dark field (HAADF) imaging in an aberration-corrected STEM. The bright-field STEM image (Fig. 3a) shows the defective structures of the GO-derived graphitic carbon. The corresponding HAADF image (Fig. 3b) clearly shows that several bright dots, corresponding to heavy atoms (Co in this case), are well dispersed in the carbon matrix. The size of these dots is in the range of 2–3 Å, indicating that each bright dot corresponds to one individual Co atom. The enlarged view of the selected region (Fig. 3c) reveals that each Co atom is centred by the light elements (C, N and/or O). Additional STEM images are provided in Supplementary Fig. 3. To probe the possible bonding between the cobalt and the light elements in the Co-NG, we performed extended X-ray absorption fine structure (EXAFS) analysis at the Co *K*-edge, using both a wavelet transform (WT) and Fourier transform. WT-EXAFS analysis is a powerful method for separating backscattering atoms that provides not only a radial distance resolution, but also resolution in the *k*-space^28^. The discrimination of atoms can be identified even when these atoms overlap substantially in *R*-space. The *k*^2^-weighted *χ*(*k*) signals (Fig. 3d) and the corresponding Fourier transforms (Fig. 3e) of the Co-NG and Co-G samples show quite similar profiles, suggesting no substantial differences in the coordination environments of the Co atoms. The existence of only one single strong shell, which is usually characteristic of amorphous or poorly crystalline materials, at ∼1.5 Å in *R*-space (Fig. 3e) is indicative of a large structural disorder around Co sites, consistent with the abundant misplacement and voids observed in the aberration-corrected STEM images. Figure 3f shows the WT contour plots of the two signals based on Morlet wavelets (*κ*=3, *σ*=1) with optimum resolution at the first shell^29^. The intensity maximum A is well-resolved for the Co-NG (3.4 Å^−1^) and Co-G (3.2 Å^−1^). Since the locations of the WT maxima are highly predictable, they allow qualitative interpretation of the scattering path origins. The WT maximum is known to be affected by the path length *R*, Debye–Waller factors *σ*^2^, energy shift Δ*E* and atomic number *Z*, and this corresponds to the same location of the maximum in the *q*-space magnitude^30^. For an isolated Co–C path (*R*=2 Å), the WT maximum at 3.2 Å^−1^ in the *q*-space magnitude showed little dependence on *R*, *σ*^2^ and Δ*E*, but it is largely affected by different *Z* (3.5 Å^−1^ for Co-N path, 4.3 Å^−1^ for Co-O path, and 6.8 Å^−1^ for Co-Co path; Supplementary Fig. 4). As a result, by comparison, the WT maximum A at 3.2 Å^−1^ for the Co-G can be associated with the Co-C path, and 3.4 Å^−1^ for the Co-N path within the Co-NG. A small difference of ∼0.1 Å^−1^ between the maxima A for the Co-NG (3.4 Å^−1^) and the calculated Co-N path (3.5 Å^−1^) might arise from the much shorter length of the actual Co-N path than 2 Å. The maximum feature B at 9.0 Å^−1^ might result from the effect of side lobes and the multiple scattering paths between the light atoms, instead of from the Co–Co path, which exhibits a maximum at 6.8 Å^−1^. The validity of the above WT-EXAFS interpretation was confirmed by a least-squares curve fitting analysis carried out for the first coordination shell of Co (Supplementary Figs 5 and 6 and Supplementary Note 1).

Taken together, the data indicate that in the Co-NG the Co is atomically dispersed in the nitrogen-doped graphene matrix and it is in the ionic state with nitrogen atoms in the cobalt's first coordination sphere. Hence, nitrogen doping of the graphene provides sites for Co incorporation.

### HER activity evaluation

**Figure 4:**
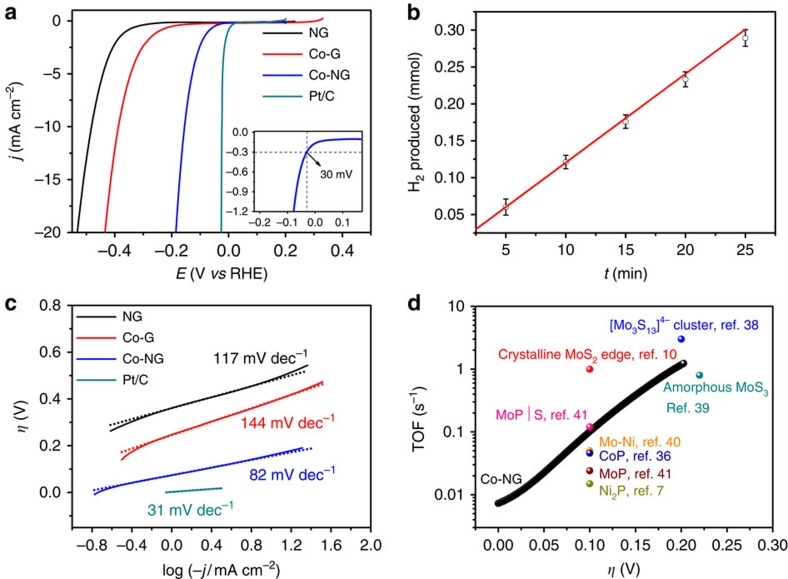
HER activity characterizations. (**a**) LSV of NG, Co-G, Co-NG and Pt/C in 0.5 M H_2_SO_4_ at scan rate of 2 mV s^−1^. The inset shows the enlarged view of the LSV for the Co-NG near the onset region. (**b**) Plot showing the molar number of H_2_ produced as a function of time. The straight line represents the theoretically calculated amounts of H_2_ assuming 100% Faradaic efficiency, and the scattered dots represent the produced H_2_ measured by gas chromatography. The overlapping of these two sets of data indicates that nearly all the current is due to H_2_ evolution. The error bars arise from instrument uncertainty. (**c**) Tafel plots of the polarization curves in **a**. (**d**) TOF values of the Co-NG catalyst (black line) along with TOF values for other recently reported catalysts.

The HER catalytic activity of the Co-NG was evaluated using a standard three-electrode electrochemical cell. The catalyst mass loading on a glassy carbon electrode was 285 μg cm^−2^. Figure 4a shows the linear-sweep voltammograms (LSVs) at a scan rate of 2 mV s^−1^ in 0.5 M H_2_SO_4_ after iR-compensation for the Co-NG electrode along with the two control samples of NG and Co-G. The commercial Pt/C (20 wt% platinum on Vulcan carbon black, Alfa Aesar) with the same mass loading was also included as a reference point. As expected, the Pt/C exhibits superior HER catalytic activity with a near zero onset *η*. The Co-NG catalyst shows excellent HER activity, as evidenced by the very small onset *η* of ∼30 mV (inset in Fig. 4a), beyond which the current density increases sharply. The onset *η* is defined here as the potential at a current density of −0.3 mA cm^−2^, which is chosen to match the onset *η* determined by the Tafel plot (shown later). The *η* needed to deliver 1 and 10 mA cm^−2^ were determined to be ∼70 and ∼147 mV, respectively. The Faradaic efficiency of the Co-NG catalyst was determined to be ∼100% by gas chromatography (Fig. 4b, Supplementary Fig. 7 and Supplementary Note 2), confirming the cathode current is due to the generation of H_2_. It should be noted that these *η* values are much smaller than those of Co-based molecular complexes^31^,^32^,^33^, and further suggesting that the Co-NG system is one of the best solid-state earth-abundant catalysts, including MoS_2_ (refs 15, 34), WS_2_ (ref. 35), CoP^36^ and MoP^37^. Also, this ‘pseudo-metal-free' catalyst (which contains only 0.57 at% metal) shows much higher activity than all the recently reported metal-free catalysts (Supplementary Table 1 and Supplementary Note 3). As control samples, the NG and Co-G show poor activity towards HER with onset *η* larger than 200 mV, indicating that the active sites in Co-NG are associated with the Co and N. Tafel analysis (Fig. 4c) gives Tafel slope values of 31, 82, 117 and 144 mV decade^−1^ for Pt/C, Co-NG, NG and Co-G, respectively. Notably, the Tafel plot for the Co-NG catalyst becomes linear at low *η* of ∼30 mV.

When tested in alkaline media (1 M NaOH), the Co-NG catalyst also exhibits improved activity compared with the NG and Co-G (Supplementary Fig. 8 and Supplementary Note 4). This distinguishes the Co-NG catalyst from the MoS_2_ and some metal phosphide (for example, Ni_2_P) catalysts, which are highly active in acid, but are unstable in base and thus their application in alkaline electrolysis is limited^2^,^7^. More interestingly, as the precursor suspension of GO containing small amounts of Co is highly stable, it can be formed into a paper (Fig. 1d), which can work as a free-standing electrode for H_2_ generation (Supplementary Movie 1). Alternatively, the precursor solution can be readily coated onto a conductive substrate (Supplementary Fig. 9 and and Supplementary Note 5) that can be used as a binder-free electrode (Supplementary Fig. 10) after post-annealing in NH_3_. The straightforward and convenient synthetic approach to achieve the Co-NG catalyst adds versatility in the design and construction of electrodes and thus enables easy integration of the catalytic layer with other components in electrochemical devices.

## Discussion

To investigate the effects of Co content on the catalytic activity, Co-NG catalysts with different Co content (from 0.03 at% to 1.23 at%, Supplementary Table 2 and Supplementary Note 6) were prepared and their HER activity were evaluated by LSV. The results (Supplementary Fig. 11 and Supplementary Fig. 12) show that HER activity does not increase linearly with the Co content, but instead there is a saturation point for Co content, beyond which the HER activity starts to decrease. This trend might be due to excess Co content; the extra Co atoms would not be able to be incorporated into the C-N lattices in graphene. Instead, the excessive Co would form Co-containing particles or clusters, such as cobalt oxide, as evidenced by the much higher oxygen content in the Co-NG sample with the highest Co content (Supplementary Table 2 and Supplementary Fig. 13). To study the effects of nitrogen doping level on the HER activity, samples with different N doping concentration were prepared by varying the annealing time (Supplementary Figs 14 and 15). The electrochemical measurements (Supplementary Fig. 16 and Supplementary Note 7) show that higher N doping level results in higher HER activity, suggesting the critical role of nitrogen in forming the catalytically active site. The influence of nitrogen doping temperature on HER activity was also studied. The results (Supplementary Fig. 17 and Supplementary Note 8) show that doping temperature above 550 °C is necessary to observe appreciably improved HER activity, which implies that the high temperature was necessary to induce Co-N interaction and thus to create Co-N-active sites. The optimized doping temperature was 750 °C with the highest N-doping level (Supplementary Table 3 and Supplementary Fig. 18). These optimizations further suggest that the HER-active sites involve the coupling effects between Co and N.

The most important figure of merit to evaluate in the intrinsic activity of a catalyst is its turnover frequency (TOF), which gives its activity on a per-site basis. To quantify the number of active sites in Co-NG, each Co centre is considered to account for one active site (see Supplementary Note 9). The contribution from the C–N matrix can be ignored as the exchange current density (*i*_0_), determined from the Tafel plot by an extrapolation method, for the NG (8.34 × 10^−7^ A cm^−2^) is much smaller than that of the Co-NG (1.25 × 10^−4^ A cm^−2^). Figure 4d shows the TOF values for the Co-NG catalyst against applied *η* together with those of eight recently reported non-precious-metal HER catalyst at specific *η*, including ultra-high vacuum (UHV)-deposited MoS_2_ nanocrystals on a Au substrate^10^, [Mo_3_S_13_]^2−^ nanoclusters supported on graphite paper^38^, amorphous MoS_3_ (ref. 39), Ni-Mo nanopowders^40^, Ni_2_P^7^, CoP^36^, MoP^41^ and MoP|S nanoparticles^41^.

At *η* of 50, 100, 150 and 200 mV, the TOF values of the Co-NG are 0.022, 0.101, 0.386 and 1.189 H_2_ s^−1^, respectively. These values reveal that the Co-NG is higher than or similar in activity to other reported catalysts, apart from the UHV-deposited MoS_2_ nanocrystals and the [Mo_3_S_13_]^2−^ nanoclusters. The TOF value of the Co-NG at thermodynamic potential (0 V vs reversible hydrogen electrode) was also calculated using the exchange current density, which gives a TOF value of 0.0054 H_2_ s^−1^. This value is approximately three times smaller than that (0.0164 H_2_ s^−1^) of the UHV-deposited MoS_2_ nanocrystals (the benchmark catalyst on MoS_2_). However, it should be noted that unlike the active site selectivity on the edge sites for MoS_2_ and on the surface sites for nanoparticulate catalysts including the amorphous MoS_3_, Ni-Mo nanopowders, Ni_2_P, CoP, MoP and MoP|S, each Co centre in our Co-NG is presumably catalytically active. To estimate the active site density (sites per cm^2^), the electrochemically active surface areas were measured (Supplementary Fig. 19), which yields an active site density of ∼9.7 × 10^13^ sites per cm^2^ (see Supplementary Note 10 for details). For comparison, Pt(111) has an active site density^10^ of 1.5 × 10^15^ sites per cm^2^.

**Figure 5:**
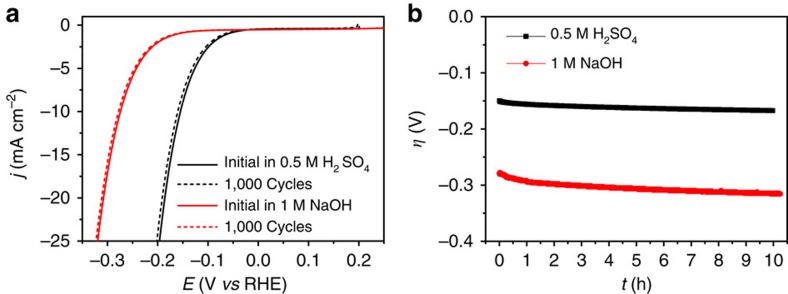
HER stability tests. (**a**) Accelerated stability measurements by recording the polarization curves for the Co-NG catalyst before and after 1,000 cyclic voltammograms at a scan rate of 50 mV s^−1^ under acidic (black curves) and basic conditions (red curves). (**b**) Plot of *η* vs *t* for the Co-NG catalyst at a constant cathodic current density of 10 mA cm^−2^ under acidic and basic conditions.

To evaluate the stability of the Co-NG catalyst, accelerated degradation studies were performed in both acid and base. As shown in Fig. 5a, the cathodic polarization curves obtained after 1,000 continuous cyclic voltammograms (scan rate: 50 mV s^−1^) shows a negligible decrease in current density compared with the initial curve, indicating the excellent stability of Co-NG in both the acid and base. In addition to the cycling tests, galvanostatic measurements at a current density of 10 mA cm^−2^ were performed and the results (Fig. 5b) show that after 10 h of continuous operation the *η* increased by 35 mV in acid and 17 mV in base, which might be associated with the desorption of some catalysts from the glassy carbon substrate during operation. The catalysts after accelerated cycling were characterized by XPS (Supplementary Figs 20 and 21), X-ray diffraction analysis (Supplementary Fig. 22) and HAADF-STEM (Supplementary Fig. 23), which suggest that cycling operation did not change the atomic Co dispersion and the chemical states of Co and N (see Supplementary Note 11 for details). The excellent stability of the Co-NG with active sites at the atomic scale can be attributed to the high-temperature-induced strong coordination between the Co and N.

In conclusion, nitrogen-doped graphene, with negligible intrinsic H_2_-evolving activity, when incorporated with very small amounts of Co as individual atoms can function as a highly active and robust HER catalyst in both acid and base media. This catalyst represents the first example of SAC achieved in inorganic solid-state catalysts for HER. This excellent catalytic performance, maximal efficiency of atomic utility, scalability and low-cost for the preparation makes this catalyst a promising candidate to replace Pt for water splitting applications. In addition, the approach demonstrated in this work in obtaining individual metal atoms that are supported on graphene may be a harbinger for broad applicability of this methodology for other atomic-scale catalytic systems.

## Methods

### Materials synthesis

All chemicals were purchased from Sigma-Aldrich unless otherwise specified. GO was synthesized from graphite flakes (∼150 μm flakes) using the improved Hummers method^42^.

### Synthesis of Co-NG

An aqueous suspension of GO (2 mg ml^−1^) was first prepared by adding 100 mg GO into 50 ml deionized water and sonicating (Cole Parmer, model 08849–00) for 2 h. One millilitre CoCl_2_·6H_2_O (3 mg ml^−1^) aqueous solution was added into the GO suspension and sonicated for another 10 min. This precursor solution was freeze-dried for at least 24 h to produce a brownish powder. The dried sample was then placed in the centre of a standard 1-inch quartz tube furnace. After pumping and purging the system with Ar three times, the temperature was ramped at 20 °C min^−1^ up to 750 °C with the feeding of Ar (150 s.c.c.m.) and NH_3_ (50 s.c.c.m.) at ambient pressure (s.c.c.m., standard cubic centimeters per minute). The reaction was allowed to proceed for 1 h and the final product Co-NG with a blackish colour was obtained after the furnace was permitted to cool to room temperature under Ar protection. The control sample of Co-G was prepared with the same treatment except NH_3_ was not introduced during the annealing process. The control sample of NG was prepared with the same treatment except that the CoCl_2_·6H_2_O was not added into the precursor solution. The Co-NG paper was fabricated by first filtering a 25-ml precursor solution (2 mg ml^−1^ GO and 0.06 mg ml^−1^ CoCl_2_·6H_2_O) through a 0.22-μm polytetrafluoroethylene membrane (Whatman). After peeling off the paper from the membrane, the cobalt-containing GO paper was annealed at 750 °C for 1 h under Ar (150 s.c.c.m.) and NH_3_ (50 s.c.c.m.) atmosphere in a tube furnace.

### Characterizations

A JEOL 6500F SEM was used to examine the sample morphology. A JEOL 2,100 field emission gun TEM was used to observe the morphologic and structural characteristics of the samples. Aberration-corrected scanning TEM images were taken using an 80-KeV JEOL ARM200F equipped with a spherical aberration corrector. Chemical compositions and elemental oxidation states of the samples were investigated by XPS spectra with a base pressure of 5 × 10^−9^ Torr. The survey spectra were recorded in a 0.5-eV step size with a pass energy of 140 eV. Detailed scans were recorded in 0.1 eV step sizes with a pass energy of 140 eV. The elemental spectra were all corrected with respect to C1s peaks at 284.8 eV. Cobalt quantitative analysis was carried using a PerkinElmer Optima 4,300 DV ICP-OES. X-ray diffraction) analysis was performed by a Rigaku D/Max Ultima II (Rigaku Corporation) configured with a CuKα radiation, graphite monoichrometer and scintillation counter. The Co *K*-edge EXAFS spectra were acquired at beamline 1W2B of the Beijing Synchrotron Radiation Facility in fluorescence mode using a fixed-exit Si(111) double crystal monochromator. The incident X-ray beam was monitored by an ionization chamber filled with N_2_, and the X-ray fluorescence detection was performed using a Lytle-type detector filled with Ar. The EXAFS raw data were then background-subtracted, normalized and Fourier transformed by the standard procedures with the IFEFFIT package^43^.

### Electrochemical measurements

The electrochemical measurements were carried out in a three-electrode setup using a CHI 608D workstation (US version). To prepare the working electrode, 4 mg of the catalyst and 80 μl of 5 wt% Nafion solution were dispersed in 1 ml of 4:1(v/v) water/ethanol with 1–2 h bath-sonication (Cole Parmer, model 08849–00) to form a homogeneous suspension. Five microlitres of the catalyst suspension were loaded onto a 3-mm-diameter glassy carbon electrode (mass loading ∼0.285 mg cm^−2^). For the counter electrode, a Pt wire was used. The reference electrode was Hg/HgSO_4_, K_2_SO_4_(sat) for measurements in 0.5 M H_2_SO_4_, and Hg/HgO, NaOH (1 M) for measurements in 1 M NaOH. Both of these two reference electrodes were calibrated against a reversible hydrogen electrode under the same testing conditions immediately before the catalytic characterizations (Supplementary Figs 24 and 25, and Supplementary Note 12). A scan rate of 2 mV s^−1^ was used in the cyclic voltammograms of the HER activity unless otherwise noted. The electrolyte solution was sparged with H_2_ for 20 min before each test.

## Additional information

**How to cite this article:** Fei, H. *et al*. Atomic cobalt on nitrogen-doped graphene for hydrogen generation. *Nat. Commun.* 6:8668 doi: 10.1038/ncomms9668 (2015).

## Supplementary Material

Supplementary Information Supplementary Figures 1-25, Supplementary Tables 1-3, Supplementary Notes 1-12 and Supplementary References

Supplementary Movie 1 Atomic cobalt hydrogen evolution

## References

[b1] Walter M. G. . Solar water splitting cells. Chem. Rev. 110, 6446–6473 (2010) . 21062097 10.1021/cr1002326

[b2] Luo J. . Water photolysis at 12.3% efficiency via perovskite photovoltaics and earth-abundant catalysts. Science 345, 1593–1596 (2014) . 25258076 10.1126/science.1258307

[b3] Jin H. . *In situ* cobalt–cobalt oxide/N-doped carbon hybrids as superior bifunctional electrocatalysts for hydrogen and oxygen evolution. J. Am. Chem. Soc. 137, 2688–2694 (2015) . 25658518 10.1021/ja5127165

[b4] Gong M. . Nanoscale nickel oxide/nickel heterostructures for active hydrogen evolution electrocatalysis. Nat. Commun 5, 4695 (2014) . 25146255 10.1038/ncomms5695

[b5] Merki D. & Hu X. Recent developments of molybdenum and tungsten sulfides as hydrogen evolution catalysts. Energy Environ. Sci. 4, 3878–3888 (2011) .

[b6] Tian J., Liu Q., Asiri A. M. & Sun X. Self-supported nanoporous cobalt phosphide nanowire arrays: an efficient 3D hydrogen-evolving cathode over the wide range of pH 0–14. J. Am. Chem. Soc. 136, 7587–7590 (2014) . 24830333 10.1021/ja503372r

[b7] Popczun E. J. . Nanostructured nickel phosphide as an electrocatalyst for the hydrogen evolution reaction. J. Am. Chem. Soc. 135, 9267–9270 (2013) . 23763295 10.1021/ja403440e

[b8] Chen W. F., Muckerman J. T. & Fujita E. Recent developments in transition metal carbides and nitrides as hydrogen evolution electrocatalysts. Chem. Commun. 49, 8896–8909 (2013) . 10.1039/c3cc44076a 23982806

[b9] Vrubel H. & Hu X. Molybdenum boride and carbide catalyze hydrogen evolution in both acidic and basic solutions. Angew. Chem. 124, 12875–12878 (2012) . 10.1002/anie.201207111 23143996

[b10] Jaramillo T. F. . Identification of active edge sites for electrochemical H_2_ evolution from MoS_2_ nanocatalysts. Science 317, 100–102 (2007) . 17615351 10.1126/science.1141483

[b11] Chen C. . Highly crystalline multimetallic nanoframes with three-dimensional electrocatalytic surfaces. Science 343, 1339–1343 (2014) . 24578531 10.1126/science.1249061

[b12] Bell A. T. The impact of nanoscience on heterogeneous catalysis. Science 299, 1688–1691 (2003) . 12637733 10.1126/science.1083671

[b13] Jiang H., Zhu L., Moon K. & Wong C. P. The preparation of stable metal nanoparticles on carbon nanotubes whose surfaces were modified during production. Carbon 45, 655–661 (2007) .

[b14] Liang Y., Li Y., Wang H. & Dai H. Strongly coupled inorganic/nanocarbon hybrid materials for advanced electrocatalysis. J. Am. Chem. Soc. 135, 2013–2036 (2013) . 23339685 10.1021/ja3089923

[b15] Li Y. . MoS_2_ nanoparticles grown on graphene: an advanced catalyst for the hydrogen evolution reaction. J. Am. Chem. Soc. 133, 7296–7299 (2011) . 21510646 10.1021/ja201269b

[b16] Liu Q. . Carbon nanotubes decorated with CoP nanocrystals: a highly active non-noble-metal nanohybrid electrocatalyst for hydrogen evolution. Angew. Chem. Int. Ed. 53, 6710–6714 (2014) . 10.1002/anie.201404161 24845625

[b17] Yang X. F. . Single-atom catalysts: a new frontier in heterogeneous catalysis. Acc. Chem. Res. 46, 1740–1748 (2013) . 23815772 10.1021/ar300361m

[b18] Kyriakou G. . Isolated metal atom geometries as a strategy for selective heterogeneous hydrogenations. Science 335, 1209–1212 (2012) . 22403387 10.1126/science.1215864

[b19] Qiao B. . Single-atom catalysis of CO oxidation using Pt_1_/FeO_x_. Nat. Chem 3, 634–641 (2011) . 21778984 10.1038/nchem.1095

[b20] Lin J. . Remarkable performance of Ir_1_/FeO_x_ single-atom catalyst in water gas shift reaction. J. Am. Chem. Soc. 135, 15314–15317 (2013) . 24090210 10.1021/ja408574m

[b21] Wei H. . FeO_x_-supported platinum single-atom and pseudo-single-atom catalysts for chemoselective hydrogenation of functionalized nitroarenes. Nat. Commun 5, 5634 (2014) . 25465918 10.1038/ncomms6634

[b22] Yang M., Allard L. F. & Flytzani-Stephanopoulos M. Atomically dispersed Au–(OH)_x_ species bound on titania catalyze the low-temperature water-gas shift reaction. J. Am. Chem. Soc. 135, 3768–3771 (2013) . 23437858 10.1021/ja312646d

[b23] Moses-DeBusk M. . CO oxidation on supported single Pt atoms: experimental and ab initio density functional studies of CO interaction with Pt atom on θ-Al_2_O_3_(010) surface. J. Am. Chem. Soc. 135, 12634–12645 (2013) . 23952672 10.1021/ja401847c

[b24] Andreiadis E. S. . Molecular engineering of a cobalt-based electrocatalytic nanomaterial for H_2_ evolution under fully aqueous conditions. Nat. Chem 5, 48–53 (2013) . 23247177 10.1038/nchem.1481

[b25] Xue Y. . Low temperature growth of highly nitrogen-doped single crystal graphene arrays by chemical vapor deposition. J. Am. Chem. Soc. 134, 11060–11063 (2012) . 22721268 10.1021/ja302483t

[b26] Ferrandon M. . Multitechnique characterization of a polyaniline–iron–carbon oxygen reduction catalyst. J. Phys. Chem. C 116, 16001–16013 (2012) .

[b27] Wu G. . Synthesis-structure-performance correlation for polyaniline-Me-C non-precious metal cathode catalysts for oxygen reduction in fuel cells. J. Mater. Chem. 21, 11392–11405 (2011) .

[b28] Funke H., Scheinost A. C. & Chukalina M. Wavelet analysis of extended x-ray absorption fine structure data. Phys. Rev. B 71, 094110 (2005) .

[b29] Funke H., Chukalina M. & Scheinost A. C. A new FEFF-based wavelet for EXAFS data analysis. J. Synchrotron Radiat. 14, 426–432 (2007) . 17717385 10.1107/S0909049507031901

[b30] Savinelli R. O. & Scott S. L. Wavelet transform EXAFS analysis of mono- and dimolybdate model compounds and a Mo/HZSM-5 dehydroaromatization catalyst. Phys. Chem. Chem. Phys 12, 5660–5667 (2010) . 20431821 10.1039/b926474d

[b31] Sun Y. . Molecular cobalt pentapyridine catalysts for generating hydrogen from water. J. Am. Chem. Soc. 133, 9212–9215 (2011) . 21612276 10.1021/ja202743r

[b32] Artero V., Chavarot-Kerlidou M. & Fontecave M. Splitting water with cobalt. Angew. Chem. Int. Ed. 50, 7238–7266 (2011) . 10.1002/anie.201007987 21748828

[b33] Cobo S. . A Janus cobalt-based catalytic material for electro-splitting of water. Nat. Mater 11, 802–807 (2012) . 22863815 10.1038/nmat3385

[b34] Xie J. . Controllable disorder engineering in oxygen-incorporated MoS_2_ ultrathin nanosheets for efficient hydrogen evolution. J. Am. Chem. Soc. 135, 17881–17888 (2013) . 24191645 10.1021/ja408329q

[b35] Cheng L. . Ultrathin WS_2_ nanoflakes as a high-performance electrocatalyst for the hydrogen evolution reaction. Angew. Chem. Int. Ed. 53, 7860–7863 (2014) . 10.1002/anie.201402315 24838978

[b36] Popczun E. J. . Highly active electrocatalysis of the hydrogen evolution reaction by cobalt phosphide nanoparticles. Angew. Chem. Int. Ed. 126, 5531–5534 (2014) . 10.1002/anie.201402646 24729482

[b37] Wang X. . Molybdenum phosphide as an efficient electrocatalyst for hydrogen evolution reaction. Energy Environ. Sci 7, 2624–2629 (2014) .

[b38] Kibsgaard J., Jaramillo T. F. & Besenbacher F. Building an appropriate active-site motif into a hydrogen-evolution catalyst with thiomolybdate [Mo_3_S_13_]^2−^ clusters. Nat. Chem. 6, 248–253 (2014) . 24557141 10.1038/nchem.1853

[b39] Merki D., Fierro S., Vrubel H. & Hu X. Amorphous molybdenum sulfide films as catalysts for electrochemical hydrogen production in water. Chem. Sci. 2, 1262–1267 (2011) .

[b40] McKone J. R., Sadtler B. F., Werlang C. A., Lewis N. S. & Gray H. B. Ni–Mo nanopowders for efficient electrochemical hydrogen evolution. ACS Catal 3, 166–169 (2012) .

[b41] Kibsgaard J. & Jaramillo T. F. Molybdenum phosphosulfide: an active, acid-stable, earth-abundant catalyst for the hydrogen evolution reaction. Angew. Chem. Int. Ed. 53, 14433–14437 (2014) . 10.1002/anie.201408222 25359678

[b42] Marcano D. C. . Improved synthesis of graphene oxide. ACS Nano 4, 4806–4814 (2010) . 20731455 10.1021/nn1006368

[b43] Newville M. IFEFFIT: interactive XAFS analysis and FEFF fitting. J. Synchrotron Radiat. 8, 322–324 (2001) . 11512767 10.1107/s0909049500016964

