# Supplementary material for: Atomic cobalt on nitrogen-doped graphene for hydrogen generation
Source: Nat Commun. 2015 Oct 21;6:8668. doi: 10.1038/ncomms9668 (PMC4639894; doi:10.1038/ncomms9668)
Supplement: Supplementary Information — Supplementary Figures 1-25, Supplementary Tables 1-3, Supplementary Notes 1-12 and Supplementary References [file ncomms9668-s1.pdf]

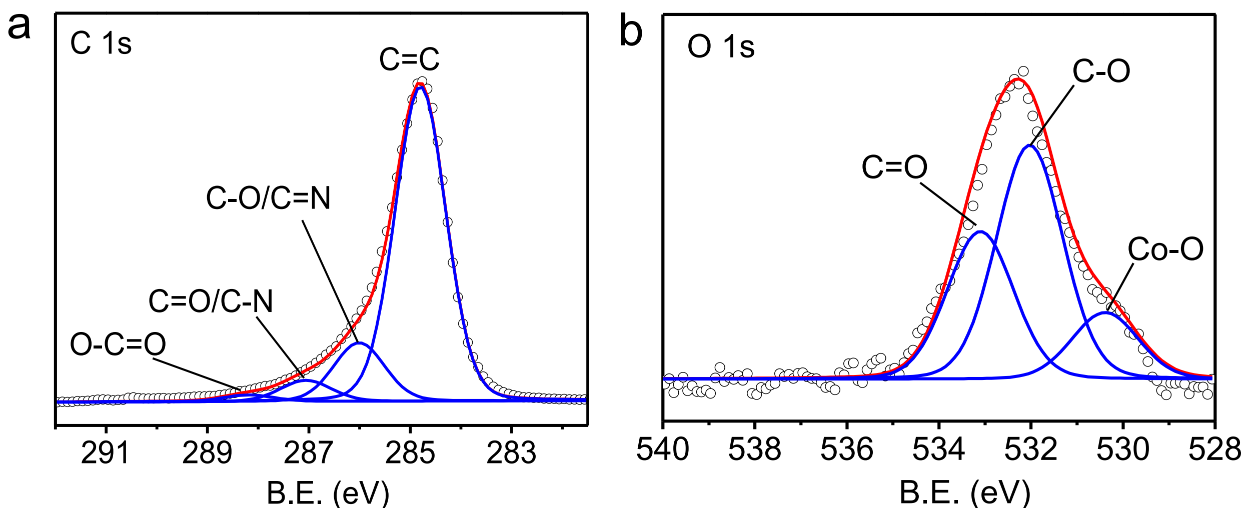

Supplementary Figure 1. XPS C 1s and O 1s spectra of the Co-NG.

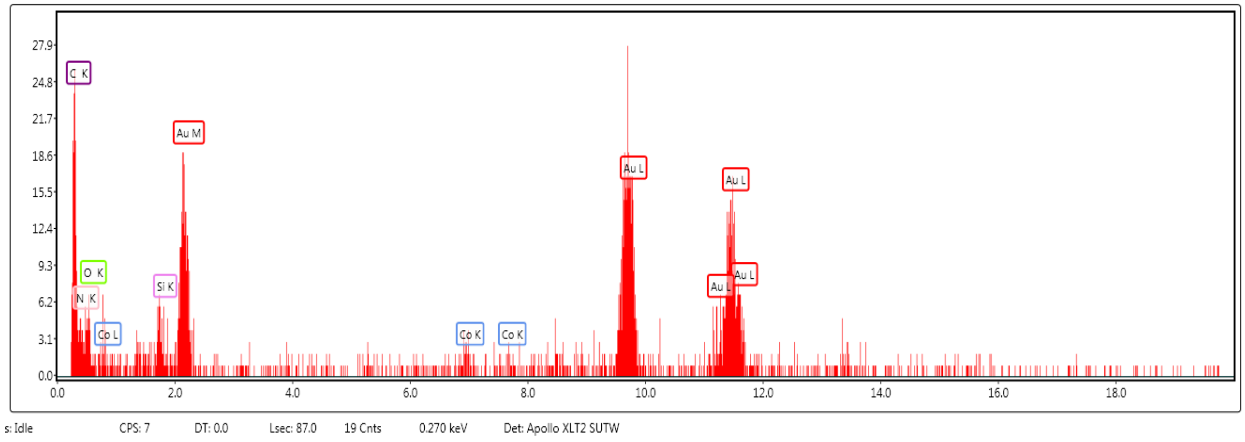

Supplementary Figure 2. STEM-EDS measurement of the Co-NG taken in the region shown in Fig. 2e. The spectrum shows the presence of the Co, N, C and O from the sample. The Au signal is from the TEM grid. The Si signal occurs from the spurious Si emission from the EDS detector.

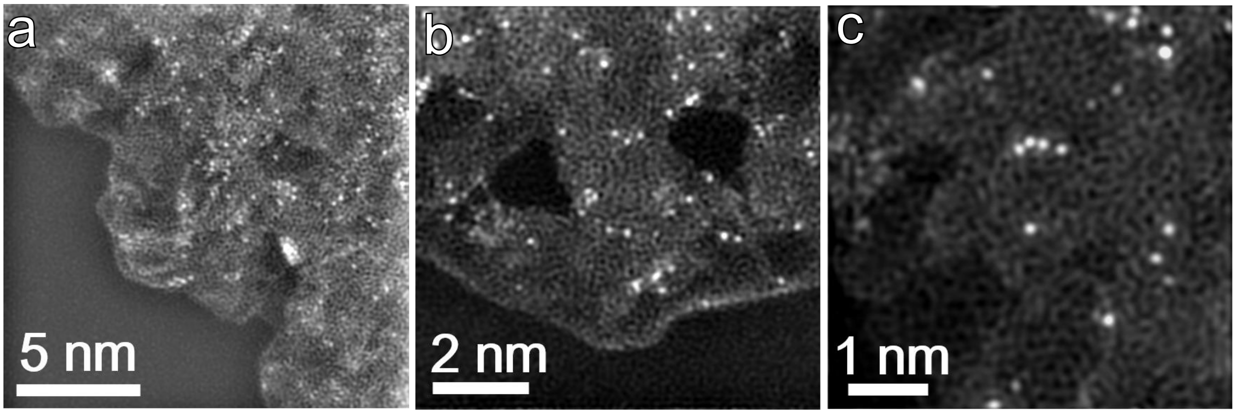

Supplementary Figure 3. Additional aberration-corrected STEM images (dark-field), showing the atomic distributions of the Co atoms. The lower magnification STEM in (a) indicates that the majority of the cobalt are isolated as individual atoms, except for some small portions of aggregated clusters.

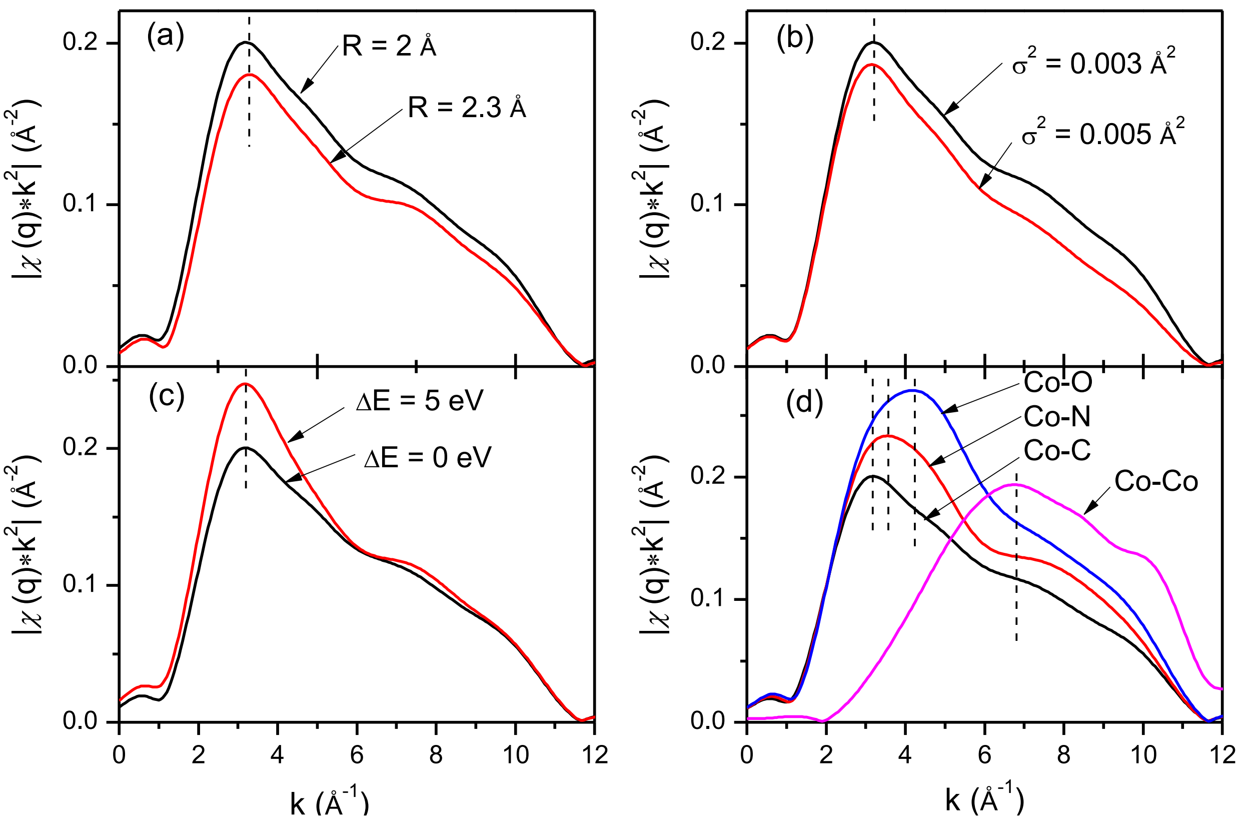

Supplementary Figure 4. Comparison of the *q*-space magnitudes for FEFF-calculated, k^2-weighted EXAFS paths. (a) The effect of the path length R on the Co-C path (with σ^2 and ΔE fixed at 0.003 Å^2 and 0 eV, respectively). (b) The effect of the Debye–Waller factors σ^2 on the Co-C path (with R and ΔE fixed at 2 Å and 0 eV, respectively). (c) The effect of the energy shift ΔE on the Co-C path (with R and σ^2 fixed at 2 Å and 0.003 Å^2, respectively). (d) The effect of atomic number Z (with R, σ^2 and ΔE fixed at 2 Å, 0.003 Å^2, and 0 eV, respectively).

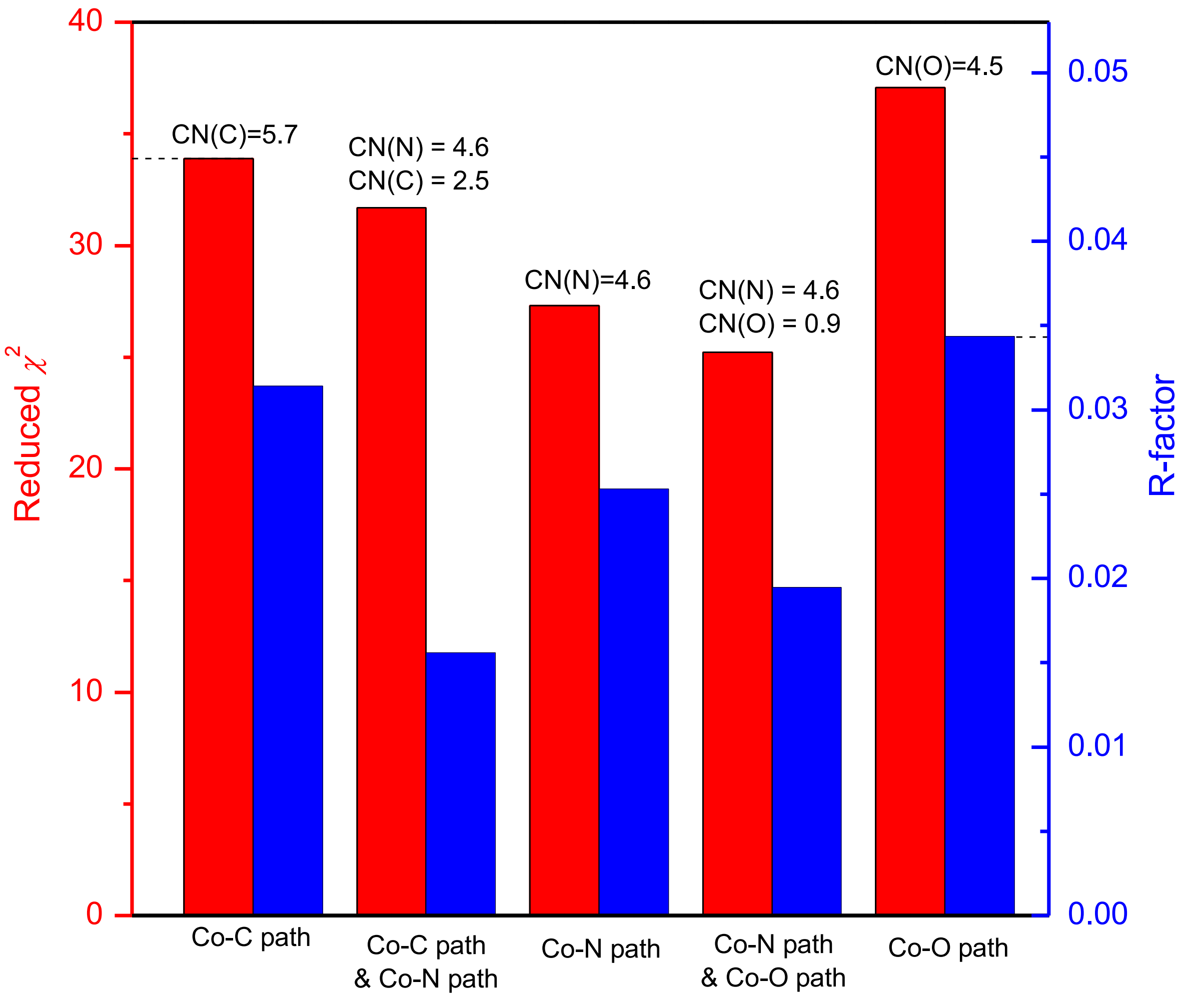

Supplementary Figure 5. The reduced χ^2 (red column) and *R*-factor (blue column) for the five structural models (the pure Co-C path, a mixture of Co-C and Co-N paths, pure Co-N path, a mixture of Co-N and Co-O paths, and pure Co-O path) used to describe the local structure of the Co-NG.

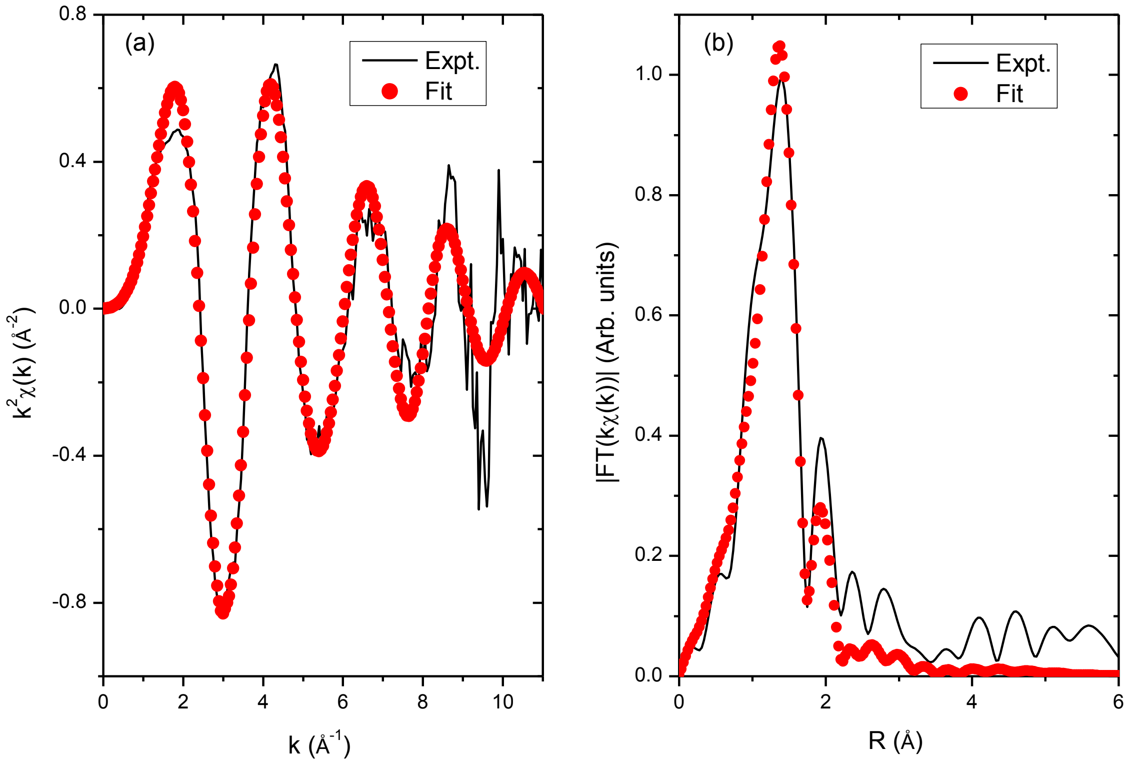

Supplementary Figure 6. Comparison between the experimental EXAFS spectrum of Co-NG and the best-fit result using the mixed model in (a) *k* and (b) *R* spaces.

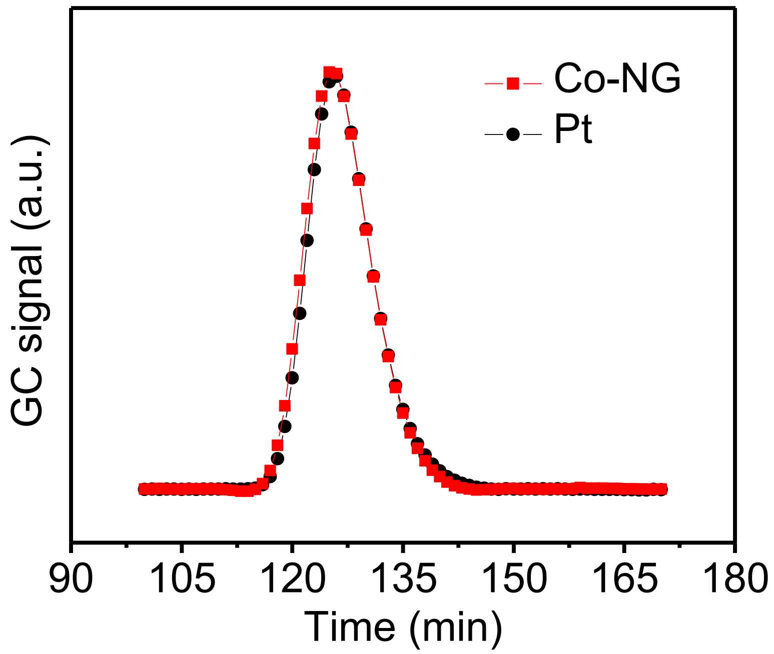

Supplementary Figure 7. GC signals for the Co-NG electrode and Pt reference electrode after 5 min reaction.

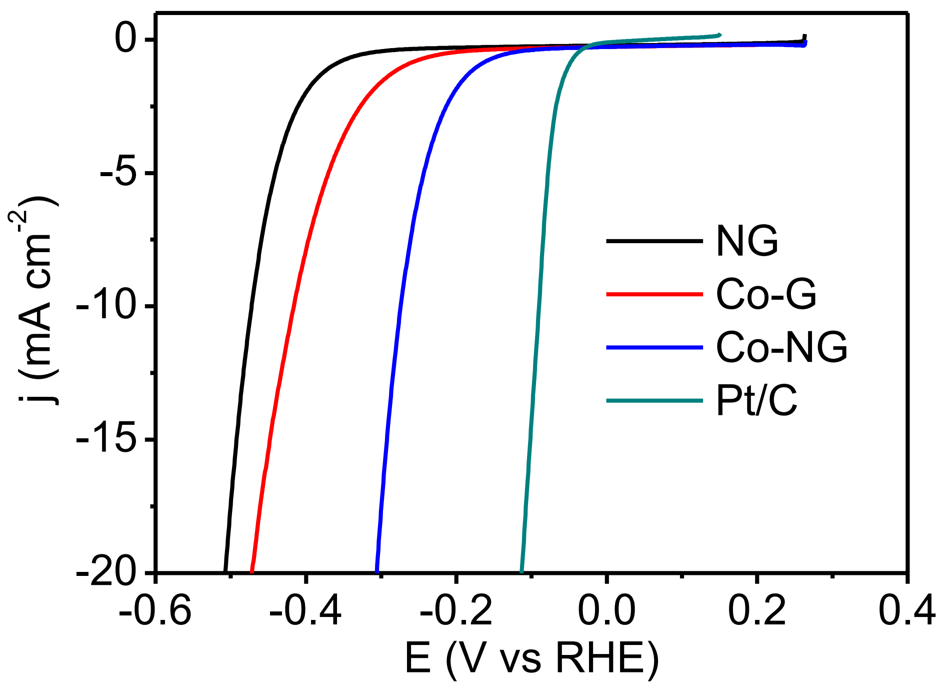

Supplementary Figure 8. Polarization curves of NG, Co-G, Co-NG and Pt/C in 1 M NaOH electrolyte at a scan rate of 2 mV s^{-1}.

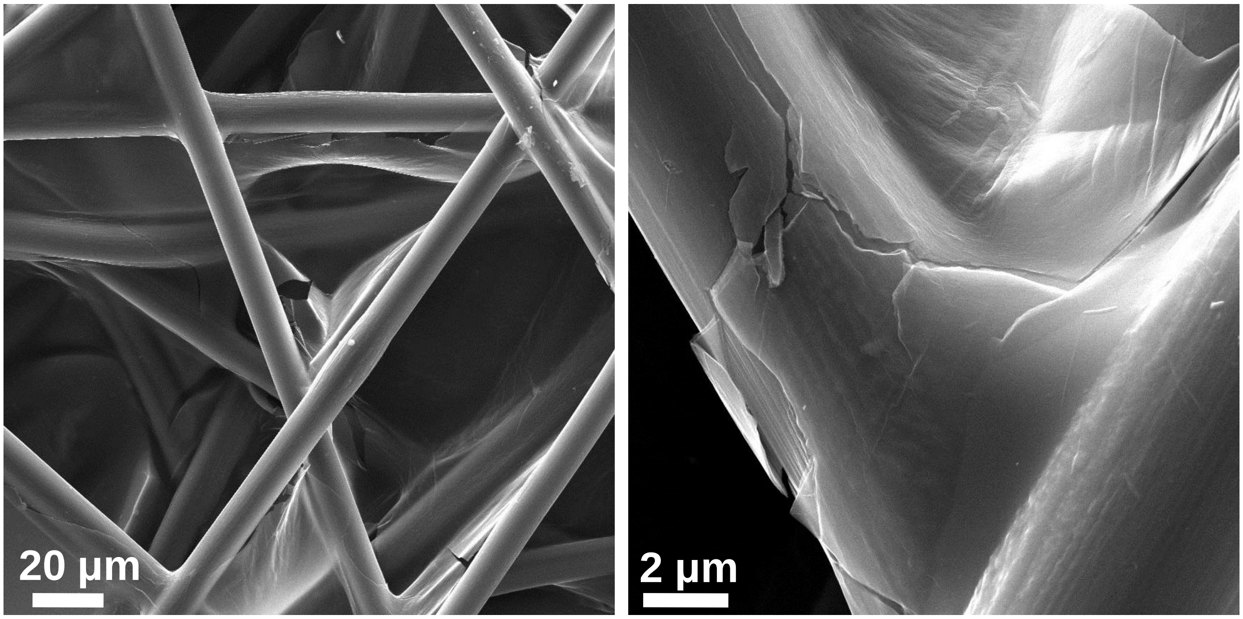

Supplementary Figure 9. SEM images of the Co-NG flakes on CFP.

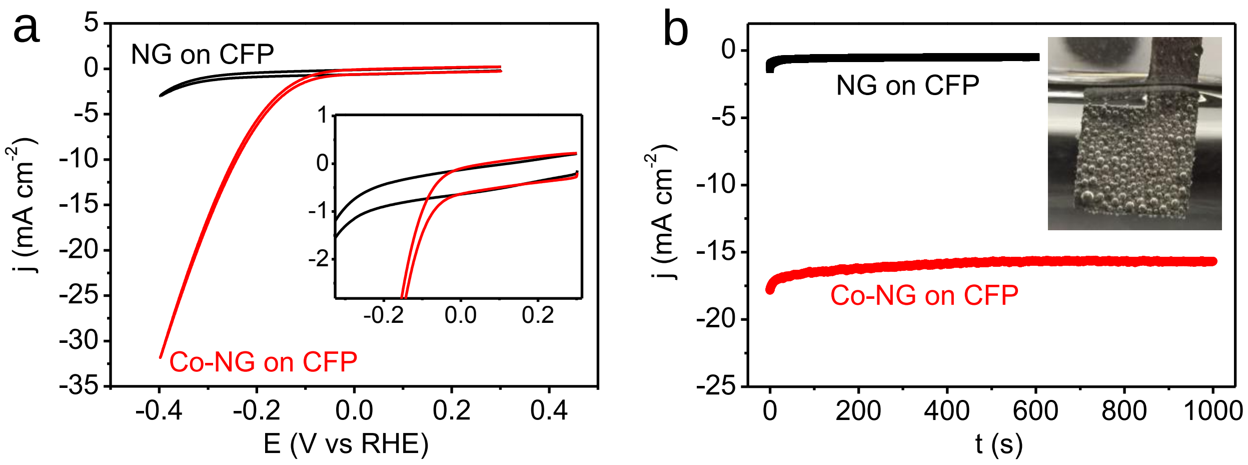

Supplementary Figure 10. (a) CV curves (0.5 M H_2SO_4, scan rate 50 mV s^{-1}, not iR-corrected) of Co-NG on CFP electrode and NG on CFP electrode. Mass loading is ~40 μg cm^{-2}. The inset shows the enlarged view near the onset region. (b) The current density versus time response at a constant η of 300 mV. The inset is the photograph of the Co-NG on the CFP electrode and shows that the surface is covered with H_2 bubbles after 30 s.

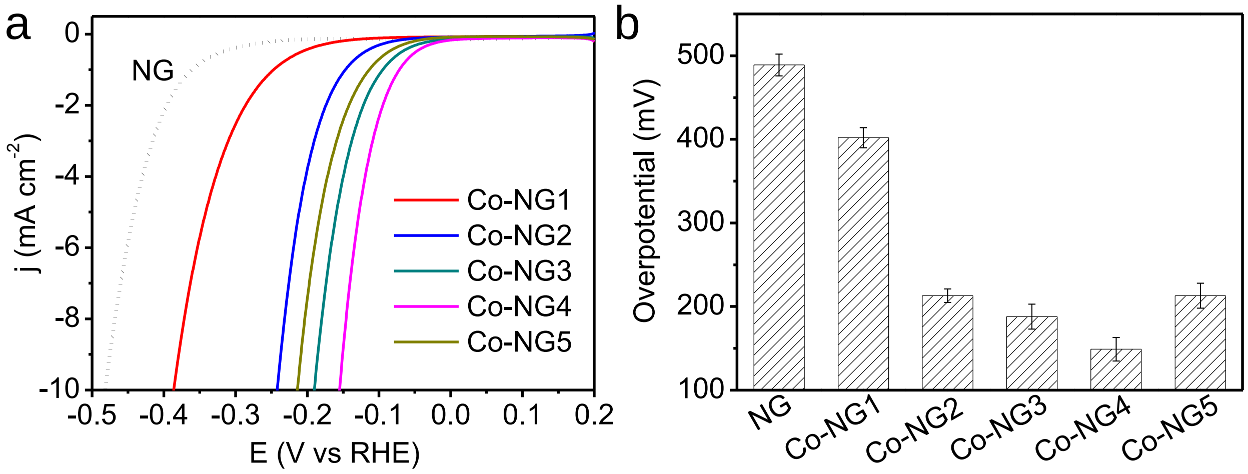

Supplementary Figure 11. (a) LSV polarization curves for the catalysts with different Co contents shown in Supplementary Table 2. (b) The η@10 mA cm^{-2} for the catalysts with different Co contents shown in Supplementary Table 2. The error bars arise from standard deviations obtained from multiple electrodes on multiple samples.

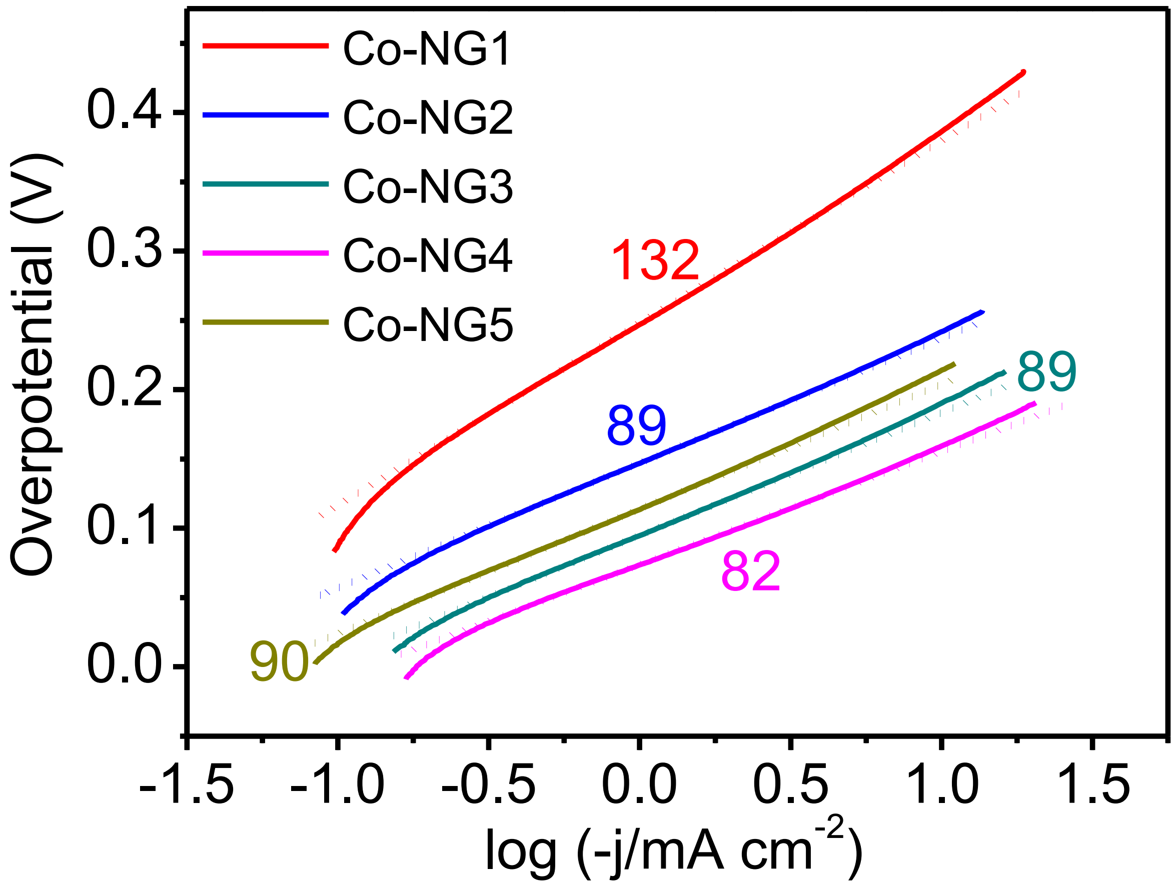

Supplementary Figure 12. Tafel plots obtained from the polarization curves for catalysts with different Co contents shown in Supplementary Table 2.

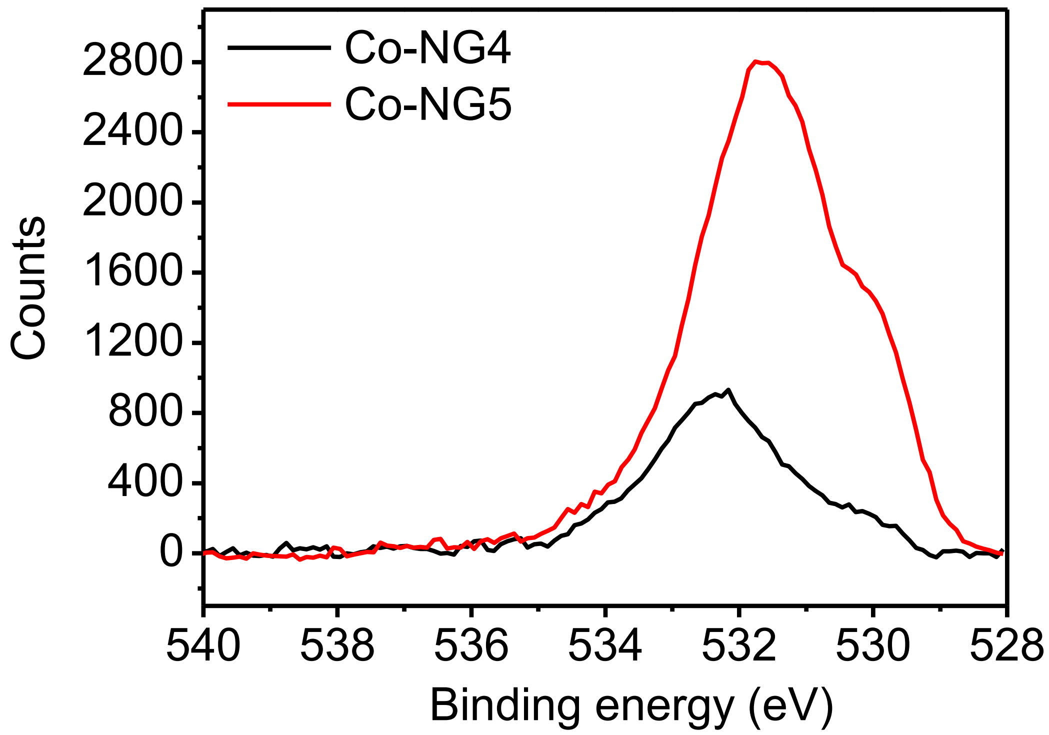

Supplementary Figure 13. O1s XPS peak for the Co-NG4 and Co-NG5.

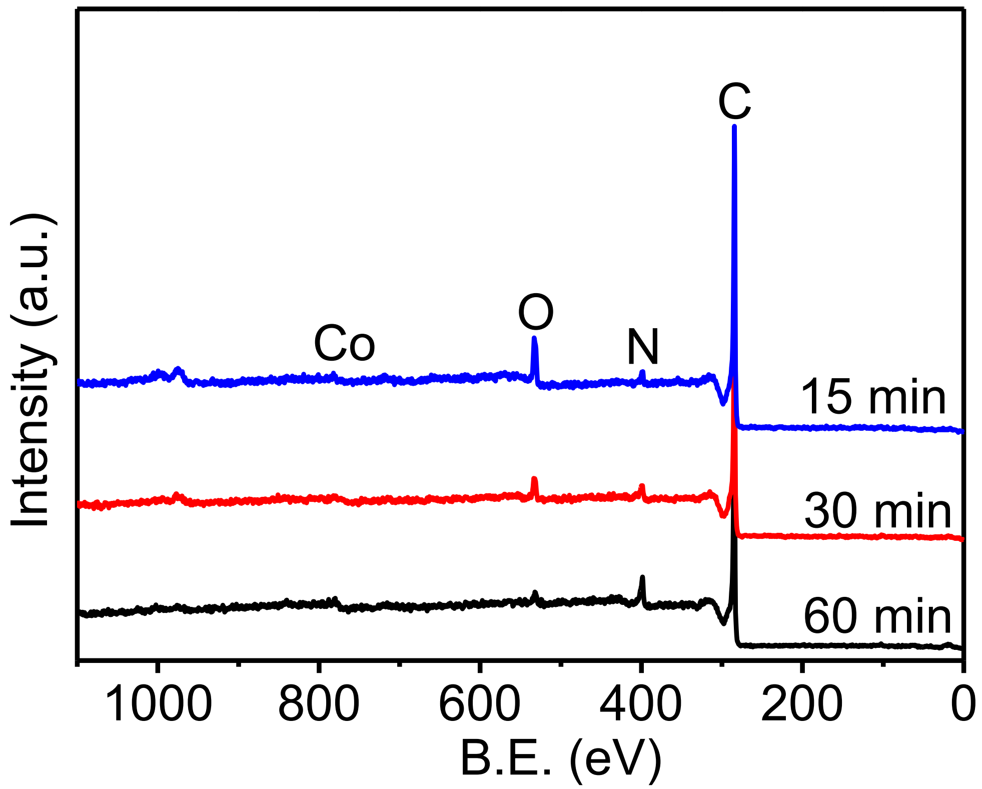

Supplementary Figure 14. XPS survey spectra for samples with different N doping concentration prepared by varying the doping time from 15 min to 60 min.

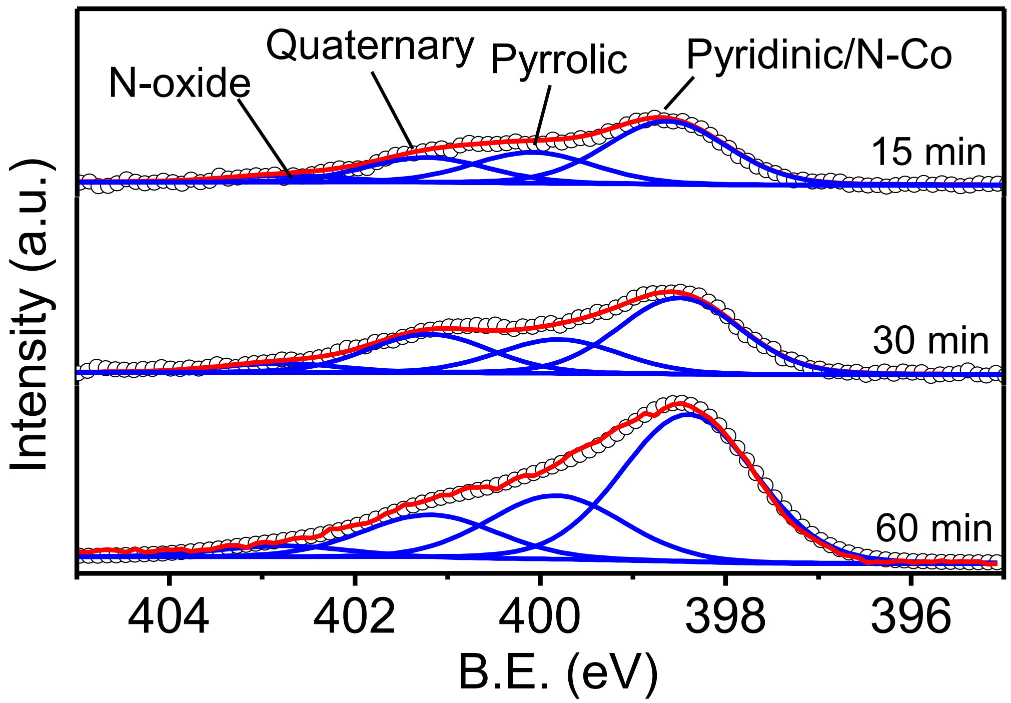

Supplementary Figure 15. XPS N 1s spectra for samples with different N doping concentration prepared by varying the doping time from 15 min to 60 min.

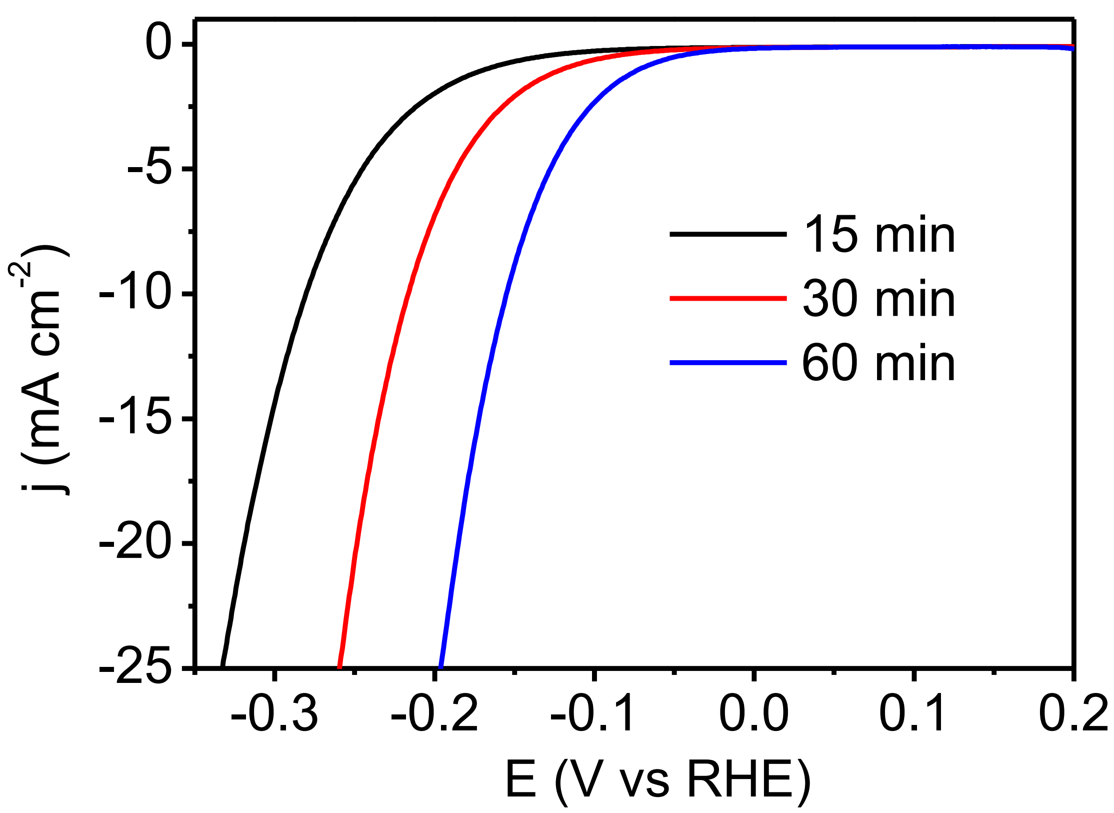

Supplementary Figure 16. LSV polarization curves for samples with different N doping concentration prepared by varying the doping time from 15 min to 60 min.

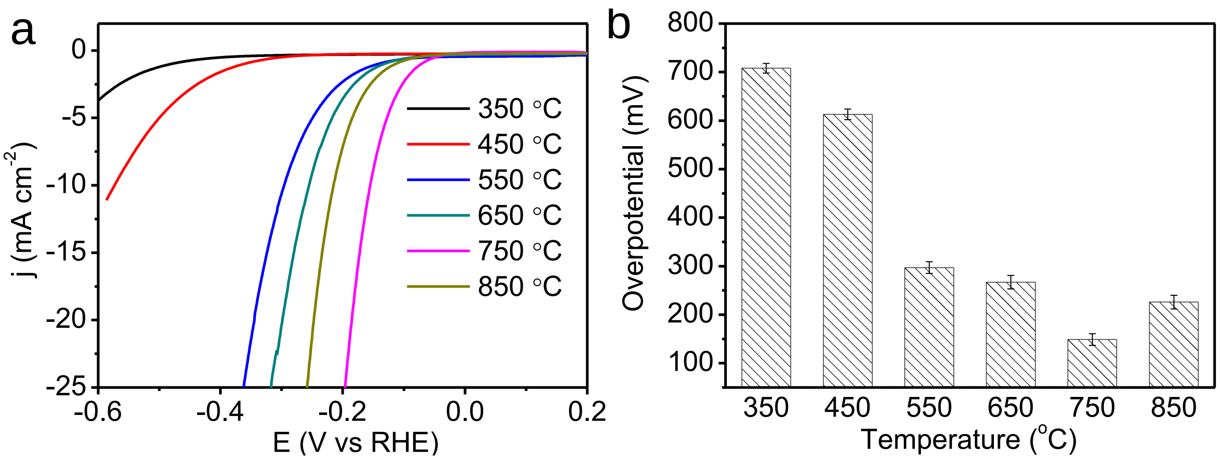

Supplementary Figure 17. (a) LSV polarization curves for the catalysts annealed at different temperatures. (b) The η@10 mA cm^{-2} for the catalysts annealed at different temperatures.

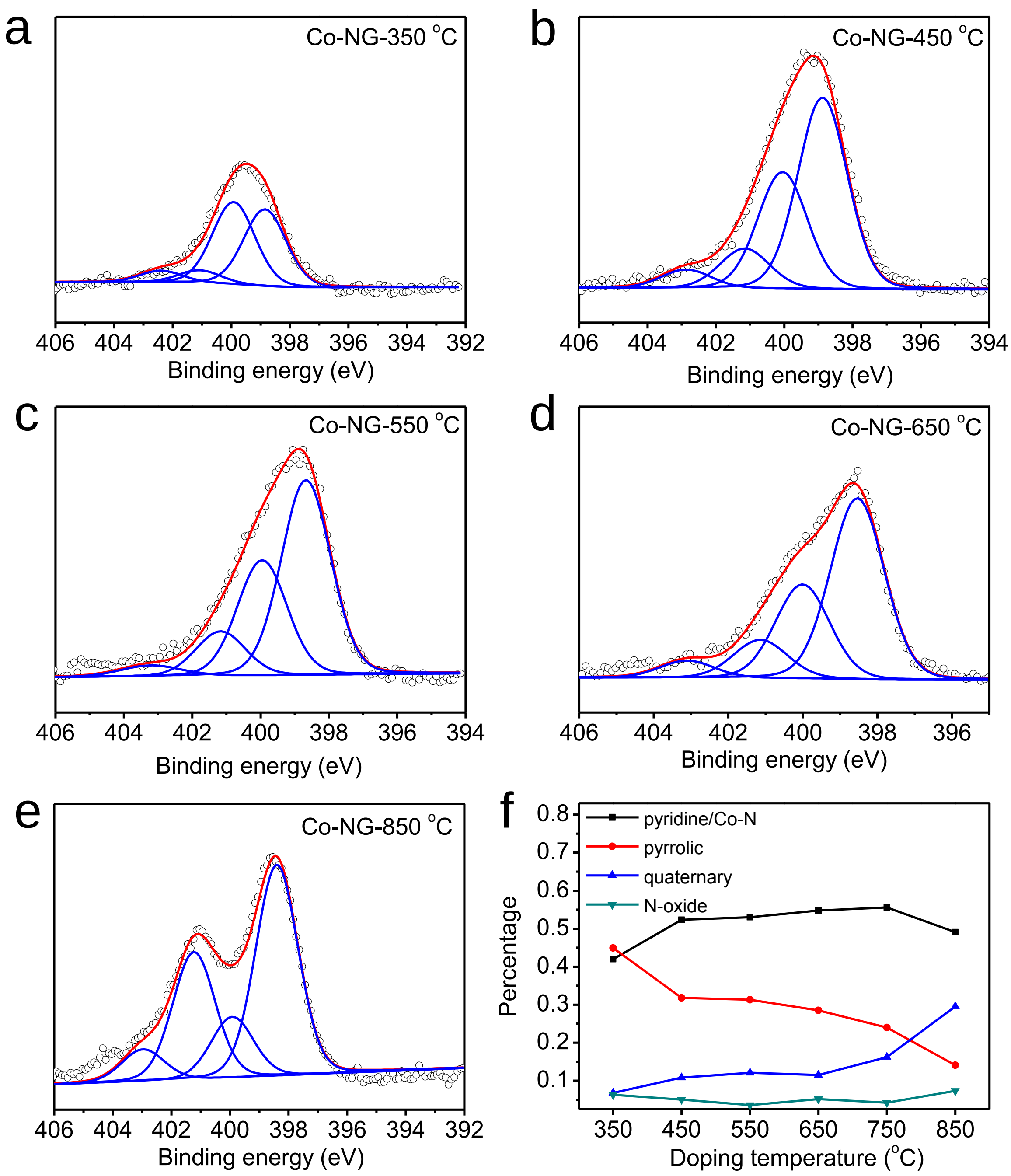

Supplementary Figure 18. The deconvoluted N1s XPS of Co-NG annealed at (a) 350 ºC, (b) 450 ºC, (c) 550 ºC, (d) 650 ºC and (e) 850 ºC. The N1s was deconvoluted into four types: pyridinic/N-Co (398.4 eV), pyrrolic (399.8 eV), graphitic (401.2 eV), and N-oxide (402.8). (f) Relationship between the percentages of the different N species and the annealing temperatures.

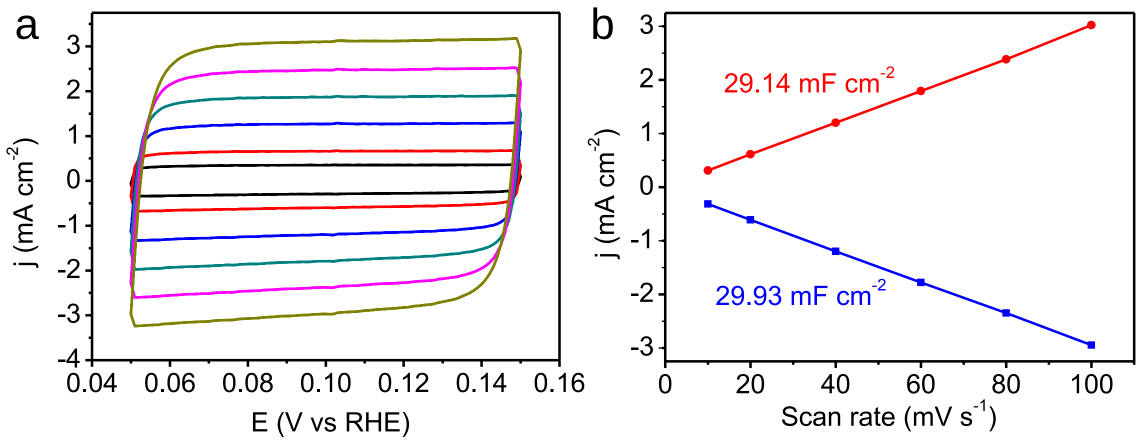

Supplementary Figure 19. Double-layer capacitance measurements for determining the electrochemical active surface area for the Co-NG with mass loading 285 μg cm^{-2}. (a) CVs measured in a non-Faradaic region at scan rate of 10 mV s^{-1}, 20 mV s^{-1}, 40 mV s^{-1}, 60 mV s^{-1}, 80 mV s^{-1}, and 100 mV s^{-1}. (b) The cathodic (red) and anodic (blue) currents measured at 0.1 V *vs* RHE as a function of the scan rate. The average of the absolute value of the slope is taken as the double-layer capacitance of the electrode.

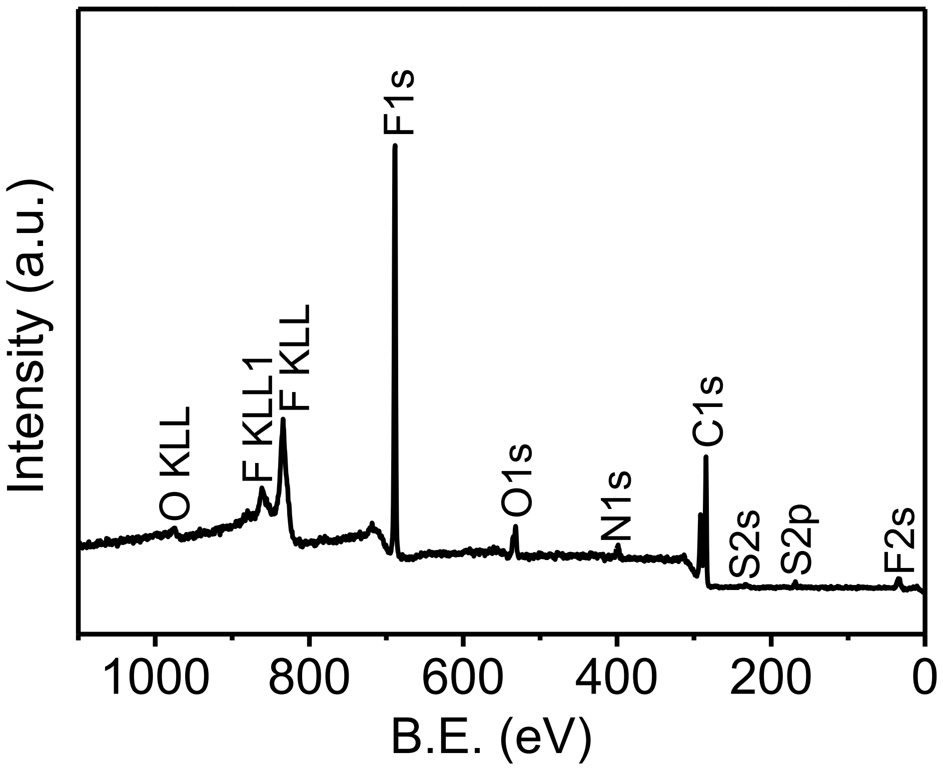

Supplementary Figure 20. XPS survey spectra of the Co-NG after cycling.

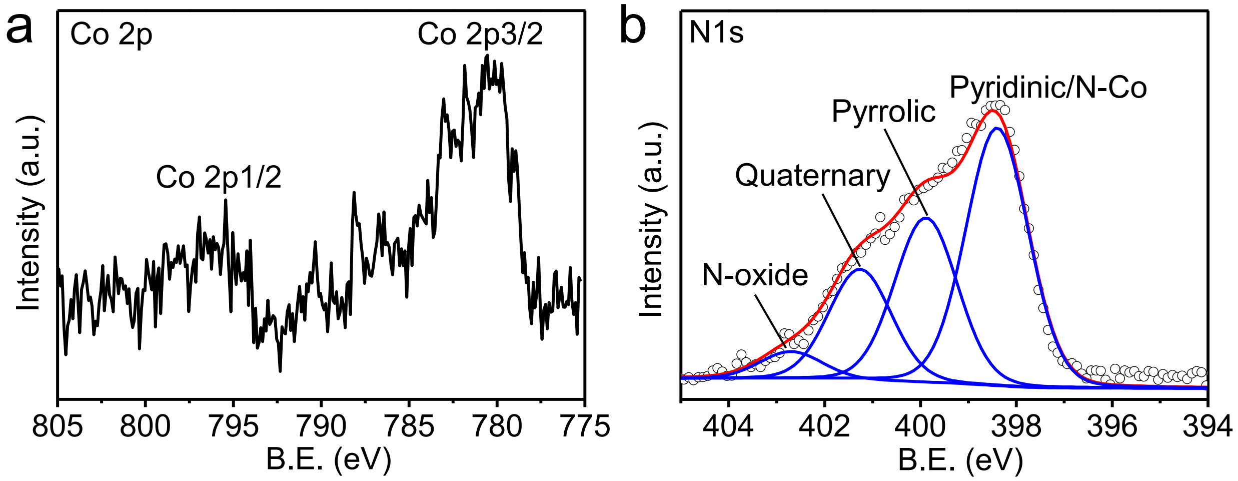

Supplementary Figure 21. (a) XPS Co 2p spectra of Co-NG after cycling. (b) XPS N 1s spectra of Co-NG after cycling.

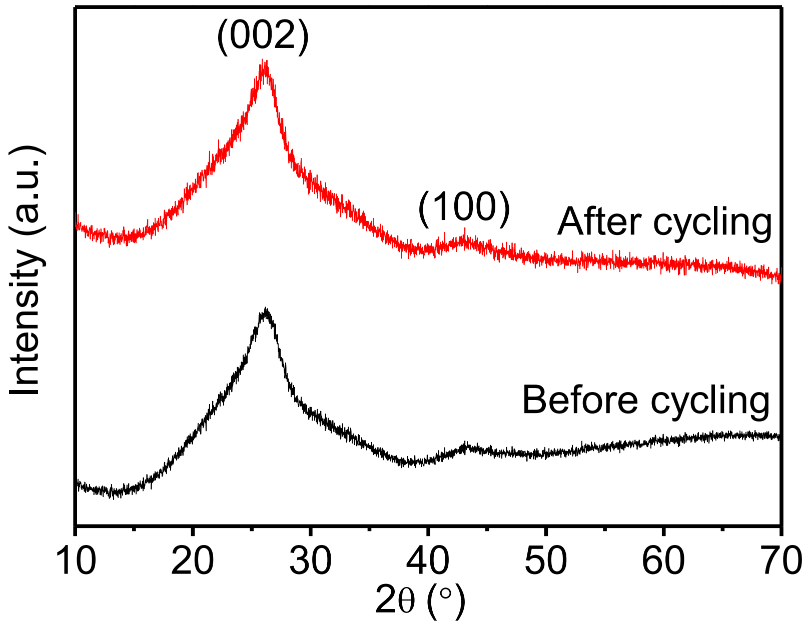

Supplementary Figure 22. XRD patterns of Co-NG before and after cycling.

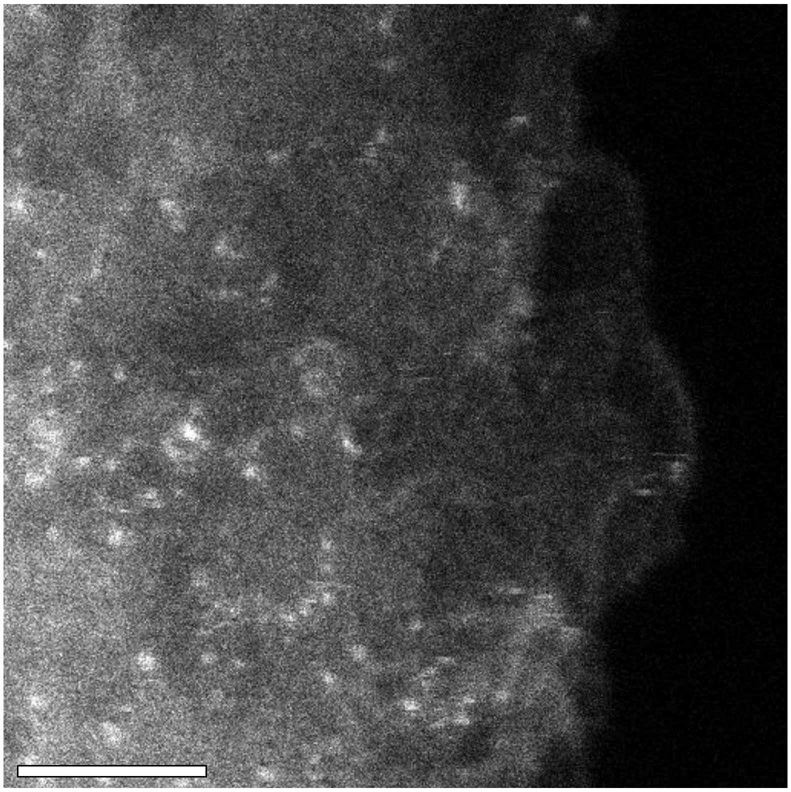

Supplementary Figure 23. HAADF-STEM image of the Co-NG after cycling.

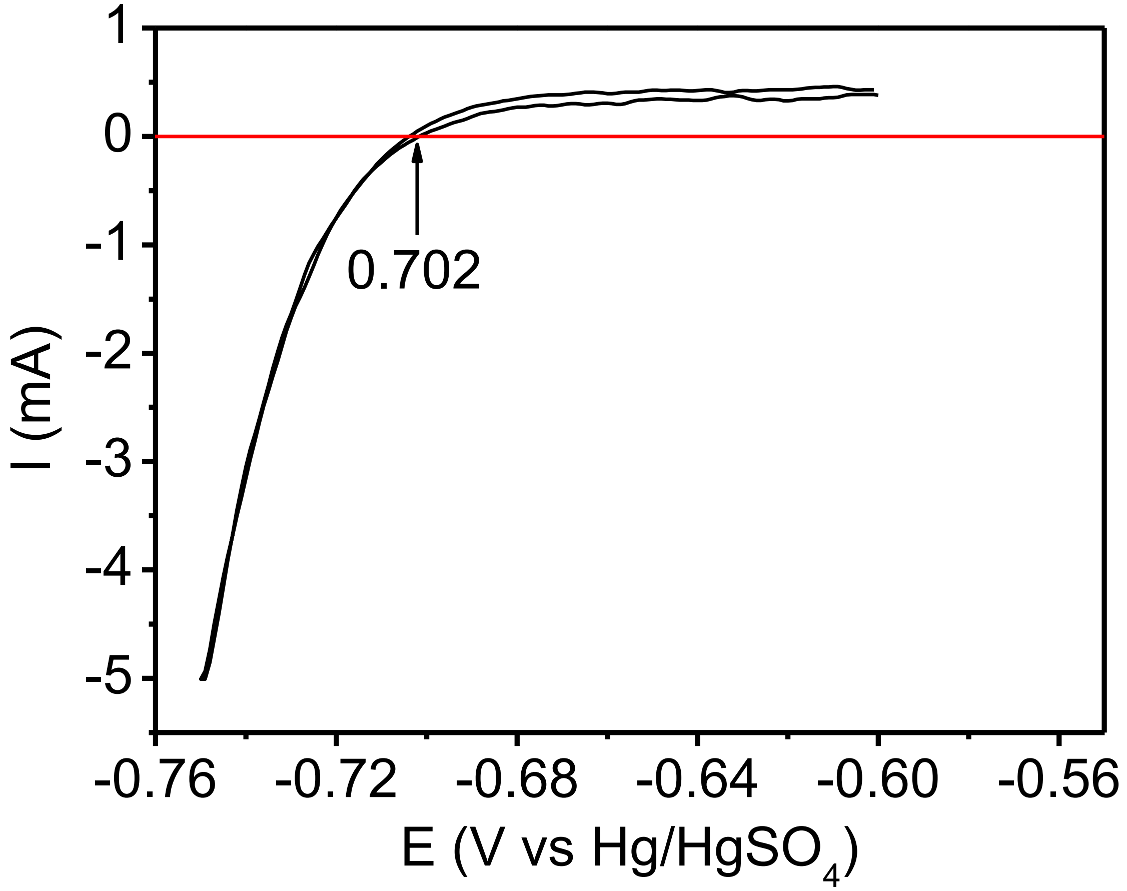

Supplementary Figure 24. Calibration of Hg/$HgSO_4$, K_2SO_4 (sat) reference electrode in 0.5 M H_2SO_4.

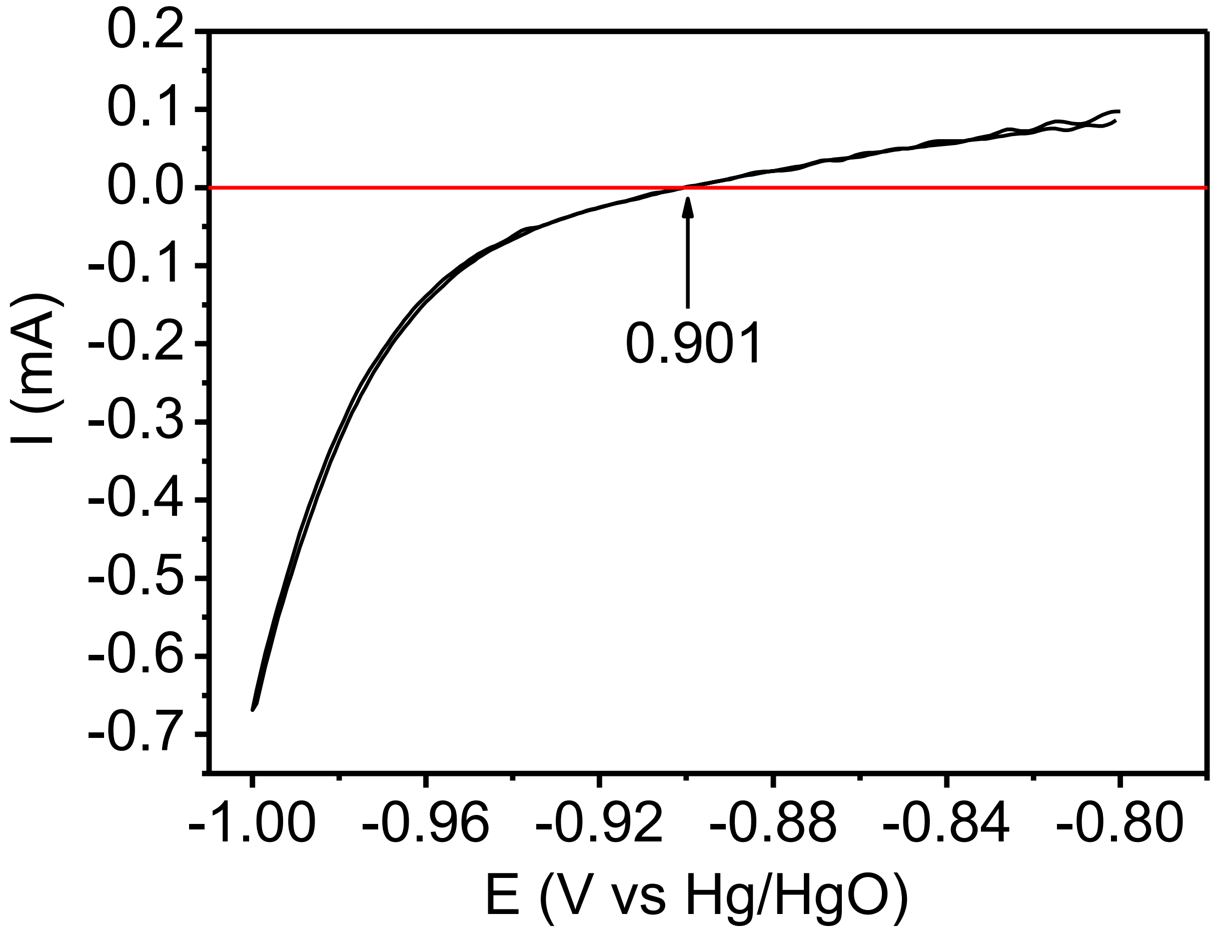

Supplementary Figure 25. Calibration of Hg/HgO, NaOH (1 M) reference electrode in 1 M NaOH.

Supplementary Table 1. Collected data of HER activity in acidic electrolyte solution.

Catalyst	Loading (mg cm^{-2})	Electrolyte	η (mV) @10 mA cm^{-2}	Ref.
Co-NG	0.285	0.5 M H_2SO_4	147	This work
$[Mo_3S_{13}]^{2-}$ clusters	0.1	0.5 M H_2SO_4	180	2
MoS_2/RGO	0.285	0.5 M H_2SO_4	150	3
Defect-rich MoS_2	0.285	0.5 M H_2SO_4	190	4
Oxygen-incorporated MoS_2	0.285	0.5 M H_2SO_4	160	5
Double gyroid MoS_2	0.06	0.5 M H_2SO_4	206	6
MoS_x| N-CNT	N/A	0.5 M H_2SO_4	110	7
1T MoS_2	0.05	0.5 M H_2SO_4	207	8
Electrodeposited amorp. MoS_3	N/A	1 M H_2SO_4	242	9
Wet-chemical amorp. $MoS_{\mathbf{x}}$	N/A	0.5 M H_2SO_4	200	10
WS_2 nanoflakes	0.35	0.5 M H_2SO_4	170	11
WS_2/rGO	0.4	0.5 M H_2SO_4	~280	12
1T WS_2	~0.0002	0.5 M H_2SO_4	200	13
CoP nanoparticles on CNT	0.285	0.5 M H_2SO_4	122	14
CoP nanoparticles	2	0.5 M H_2SO_4	~75	15
MoP nanoparticles	0.36	0.5 M H_2SO_4	125	16
MoS|P	1	0.5 M H_2SO_4	81	17
Ni_2P nanoparticles	1	0.5 M H_2SO_4	~100	18
MoB	2.5	1 M H_2SO_4	215	19
Mo_2C	1.4	1 M H_2SO_4	215	20
Co-NRCNTs	0.28	0.5 M H_2SO_4	260	21
FeCo@NCNTs-NH	0.32	0.5 M H_2SO_4	290	21
N- and P-doped graphene	0.2	0.5 M H_2SO_4	422	22
C_3N_4@NG	0.1	0.5 M H_2SO_4	240	23
C_3N_4 nanoribbons on graphene	0.143	0.5 M H_2SO_4	207	24
N- and S-doped graphene	N/A	0.5 M H_2SO_4	276	25
N-doped mesoporous graphene	0.57	0.5 M H_2SO_4	239	26

Notes: the reported catalysts from ref. 22 to ref. 26 are metal-free HER electrocatalysts, and all the others are based on non-precious metals.

Supplementary Table 2. Elemental compositions of the samples with different Co contents prepared by varying the volume of $CoCl_2$ solution added into the GO precursor solution.

Sample	$CoCl_2$ (μL)	Co (wt%)	Co (at%)	C (at%)	N (at%)	O (at%)
NG	0	0.0171	<0.005	89.2	7.1	3.7
Co-NG1	50	0.1356	0.03	88.9	6.5	4.5
Co-NG2	150	0.4413	0.09	87.6	7.2	4.8
Co-NG3	500	1.3238	0.29	87.1	8.0	4.7
Co-NG4	1000	2.4806	0.57	88.1	8.5	2.9
Co-NG5	2000	4.9032	1.23	81.3	7.4	10.1

Supplementary Table 3. Elemental compositions of the samples annealed at different temperatures.

Temperature (ºC)	C (at%)	N (at%)	O (at%)
350	82.6	3.1	14.3
450	84.9	5.3	9.8
550	85.8	7.0	7.2
650	88.1	7.9	4.0
750	88.6	8.5	2.9
850	92.3	4.3	3.4

Supplementary Note 1: Least-squares curve fitting analysis on EXAFS measurements

To examine the validity of the above WT-EXAFS interpretation, a least-squares curve fitting analysis was carried out for the first coordination shell spreading from 0.8 to 2.5 Å. All backscattering paths were calculated based on the structures provided by *ab initio* simulations. The amplitude reduction factor (S_0^2) was fixed at 0.96. The energy shift (ΔE_0) was constrained to be the same for all scatters. The path length R, coordination number (CN), and Debye–Waller factors σ^2 were left as free parameters. The fit was done in R space with k range of 1.5–10.5 $Å^{-1}$ and k^2 weight. Five structural models, i.e., the pure Co-C path, a mixture of Co-C and Co-N paths, pure Co-N path, a mixture of Co-N and Co-O paths, and pure Co-O path, were used to describe the local structure of the Co-NG. Both reduced χ^2 and R-factor were used as relevant parameters to determine the goodness-of-fit. As shown in Supplementary Fig. 5, a mixture of Co-N and Co-O coordinations with CN(N)=4.6 and CN(O)=0.9 provides the best fit, in good agreement with the results obtained by WT-EXAFS analysis. A comparison between the experimental spectrum and the best-fit result is shown in Supplementary Fig. 6. Thus, it can be concluded that the Co atoms in the Co-NG are preferentially bonded to the nitrogen atoms. Also, it is worth mentioning here that the Co atoms adopt a higher coordination number with nitrogen than the Fe-N bonding in a previously reported carbon nanotube–graphene complex which has a Fe–N/O coordination number of 3.3–3.6.[1]

Supplementary Note 2: Faradaic efficiency measurements

To measure the Faradaic efficiency of the Co-NG catalyst, H_2 production was performed in a closed pyrex glass reactor at a constant cathodic current density of 20 mA cm^{-2}. Continuous gas flow inside the whole reaction line was maintained by using a circulation pump. Quantitative analysis of produced H_2 was measured by gas chromatography (GC) (GOW-MAC 350) using a thermal conductivity detector (TCD). A defined amount of sampling gas was injected into the GC using a 6-port injection valve. The plot in Fig. 4b shows a good correlation between the calculated and measured amounts of H_2 gas, indicating near 100% efficiency. The production of H_2 gas was further confirmed by comparison of the GC signals of H_2 from the Co-NG and a Pt wire working electrode, which shows almost the same H_2 production activity after the same reaction time period (Supplementary Fig. 7).

Supplementary Note 3: Activity comparisons to other reported HER electrocatalysts in acid

To compare the HER activities of the Co-NG catalyst with other reported non-precious-metal catalysts and metal-free catalysts, we chose the overpotential required to deliver current density of 10 mA cm^{-2} (η@10 mA cm^{-2}) as the main parameter for comparison. Though the onset η is a good indicator on the intrinsic activity, it was not used here for comparison because of the ambiguity in determining its value. The summarized comparison data was shown in Supplementary Table 1. The activity of the Co-NG is higher than most of the Mo-based and other transition-metal based catalysts as well as all the metal-free catalysts, but slightly lower than the metal phosphide catalysts, taking the catalyst mass loadings into considerations. The

activity of the Co-NG might be further improved by adjusting the morphologic features of the graphene nanosheets (e.g. by creating nanoporosity).

Supplementary Note 4: HER activity in alkaline electrolyte

The HER activity of the Co-NG catalyst was tested in 1 M NaOH electrolyte. The control samples of Co-G and NG were also tested under the same conditions. The commercial Pt/C was included as a reference point. The testing results (Supplementary Fig. 8) show that the Co-NG has a much higher HER activity with a more positive onset η and a larger current density compared to the Co-G and NG. The activity trend of these four samples in alkaline condition is the same as that in acid. η of ~170 mV and ~270 mV are needed for the Co-NG to deliver 1 mA cm^{-2} and 10 mA cm^{-2}, respectively.

Supplementary Note 5: Co-NG catalysts on carbon fiber paper

Due to the very low content of Co, the Co-containing precursor solution, shown in Fig. 1a, can form a stable suspension similar to the pure GO solution. Benefited from this feature, the precursor solution can be easily coated on conductive substrates by straightforward methods, such as drop-coating, dip-coating or spin-coating. The coated substrates, after post-annealing treatment, can be directly used as a highly active HER electrode without using any binder. As a proof of concept, 50 μL Co-containing GO precursor solution was drop-cast onto a carbon fiber paper (CFP, from Fuel Cell Store, 2050-A) in a defined area (1×1 cm^2). This gives a precursor loading of ~100 μg cm^{-2}. The Co-NG on CFP was obtained by annealing the coated CFP under NH_3 atmosphere at 750 ºC for 1 h (same procedure as used for the preparation of Co-NG catalyst

in powder form). The active material Co-NG mass loading was estimated to be ~40 μg cm^{-2}, assuming 60 wt% loss from the GO due to thermal reduction based on the observation during preparation of the powder-form of the Co-NG catalyst.

Supplementary Fig. 9 shows SEM images of the Co-NG on CFP, which shows that the CFP is surface-wrapped with Co-NG flakes and they are in good physical contact. The Co-NG on CFP was then directly used as a working electrode for HER testing. For comparison, NG on a CFP electrode was also tested. The CV curves (Supplementary Fig. 10a) show that Co-NG on CFP has a much higher HER activity with larger current density and more positive onset η than the NG on CFP. These observations are consistent with those for powder-form catalysts, indicating the generality in preparing the Co-NG catalyst. Supplementary Fig. 10b shows the current density versus time response at a constant η of 300 mV. The Co-NG on CFP delivers a stable current during the testing period, indicating a good adhesion of the Co-NG flakes on the CFP. The initial loss of current density results from the accumulation of H_2 bubbles on the electrode, blocking some active sites. The inset photograph (taken after a 30 s chronoamperometry measurement) shows that the Co-NG on CFP electrode is covered fully with evolved H_2 bubbles. In comparison, the bubbles can be barely seen in the NG on CFP electrode (not shown) during the same time period.

Supplementary Note 6: Co contents on the influence of the HER activity of Co-NG

To investigate the influence of Co content on the HER activity, the Co-NG catalysts with different Co content were prepared by varying the amount of $CoCl_2$ added into the precursor solution, with all the other synthetic treatments kept the same. The elemental compositions of the

corresponding samples were summarized in Supplementary Table 2. The Co contents were determined by ICP-OES, and the C, N and O contents were determined by XPS. It can be seen that the Co content in the NG sample without intentionally adding Co is negligible (< 0.005 at%). The Co contents, as expected, increase linearly with the amount of $CoCl_2$ solution added. The N doping contents are in a similar range (~6 to ~8 at%) in these samples. The O contents are in the range of ~3 to ~5 at% in all the samples except for the sample Co-NG5 with the largest amount of Co, which has much higher O content of ~10 at%. Supplementary Fig. 13 shows the O1s XPS peak of the Co-NG4 and Co-NG5. Compared to the Co-NG4, the O1s peak for Co-NG5 has large portions from a lower binding energy that can be assigned to metal oxide,[27] which indicates the formation of cobalt oxide particles or clusters in the Co-NG5.

The HER activity of these samples were investigated in 0.5 M H_2SO_4. Supplementary Fig. 11a shows the LSV polarization curves of the corresponding samples in Supplementary Table 2. The results show that all the samples with the adding of Co have higher HER activity than bare NG. Co content as low as 0.09 at% (Co-NG2) is already sufficient to significantly increase the HER activity compared to NG. The activity increase continues up to 0.57 at% Co (Co-NG4), after which the activity starts to drop at 1.23 at% Co (Co-NG5). The changes of HER activity with the increase of the Co content are more clearly revealed by the η@10 mA cm^{-2} for each sample (Supplementary Fig. 11b). Analyzing the Tafel plots of these Co-NG samples yields Tafel slope values in the range of 82 to 132 mV decade^{-1} (Supplementary Fig. 12). The variation of the Tafel slope values may reflect the changes in HER mechanism in samples with different Co contents, as the active sites in the catalyst with higher Co content are more likely to be in closer proximity, which could affect the bonding of the intermediates during HER process.

Supplementary Note 7: Nitrogen-doping level on the influence of the HER activity of Co-NG

Samples with different nitrogen doping levels were prepared by varying the doping time. For example, 15 min doping time gives 3.2 at% N, 30 min gives 5.3 at% N and 60 min gives 8.5 at% N. Further increase in doping time results in no gain in N doping level, indicating 8.5 at% N is the saturation doping level. The XPS characterization (Supplementary Fig. 14 and Supplementary Fig. 15) on these three different samples show similar peak features but with shorter annealing time resulting in smaller N peak intensities. The electrochemical measurements (Supplementary Fig. 16) show that the sample with 8.5 at% N has the highest activity and the drop in N doing level leads to the decrease in HER activity.

Supplementary Note 8: Nitrogen-doping temperature on the influence of the HER activity of Co-NG

To investigate the influence of annealing temperature on the HER activity, a series of Co-NG catalysts were prepared by varying the nitrogen-doping temperature from 350 ºC to 850 º C. The C, N and O contents in these samples were determined by XPS and shown in Supplementary Table 3. The Co content is kept the same and not included. The C content was increased linearly as the doping temperature was increased. At the same time, the O content approximately followed a decreasing trend, indicating a higher degree of reduction at higher temperature. The N can be successfully doped into the GO at a temperature as low as 350 ºC with 3.1 at% N and the N content kept increasing up to 8.5 at% at 750 ºC. Further increase in doping temperature resulted in a lower N doping level. The XPS N 1s peak can be deconvoluted into different types

of N species, as has been shown in Fig. 2. Similarly, the percentages of the N species in these samples can be obtained by deconvolving the N 1s peaks and the results are shown in Supplementary Fig. 18. As the temperature is increased, there was a decreasing trend for pyrrolic N and an increasing trend for quaternary N species, indicating that the quaternary N is the stable species at high temperatures. The pyridine/Co-N species are the dominant species at high temperatures.

The HER activity of these samples were investigated in 0.5 M H_2SO_4. Supplementary Fig. 17a and Supplementary Fig. 17b show the polarization curves and the η@10 mA cm^{-2} for all the samples, respectively. The samples prepared at 350 ºC and 450 ºC show very poor HER activity with onset η larger than ~300 mV and the η@10 mA cm^{-2} are well above 600 mV, compared to ~480 mV needed for NG. There is a sudden increase in the HER activity when the annealing temperature was increased to 550 ºC, at which the η@10 mA cm^{-2} drops sharply to below 300 mV. This suggests that a high temperature (> 550 ºC) is necessary to induce the coupling or coordination between the Co and N atoms in the Co-NG catalyst. The HER activities keep increasing up to 750 ºC and then start to decrease at 850 ºC, at which temperature the N content is only 4.3 at%.

Supplementary Note 9: Turnover frequency (TOF) calculations

The per-site TOF value was calculated according to the following equation:

$$\mathrm{TOF}\ (\mathrm{H_2/s}) = \frac{\#\ \text{total hydrogen turnovers per geometric area}}{\#\ \text{active sites per geometric area}} \qquad (1)$$

The number of total hydrogen turnovers was calculated from the current density extracted from the LSV polarization curve according to:[10,17]

$$\# \text{ total hydrogen turnovers} = \left(|j|\frac{\text{mA}}{\text{cm}^2}\right)\left(\frac{1\text{C/s}}{1000\text{ mA}}\right)\left(\frac{1\text{ mol e}^-}{96485.3\text{ C}}\right)\left(\frac{1\text{ mol}}{2\text{ mol e}^-}\right)\left(\frac{6.022\times10^{23}\text{ molecules H}_2}{1\text{ mol H}_2}\right) = 3.12\times10^{15}\ \frac{\text{H}_2\text{/s}}{\text{cm}^2}\ \text{per}\ \frac{\text{mA}}{\text{cm}^2} \qquad (2)$$

The number of active sites in Co-NG catalyst was calculated from the mass loading on the glassy carbon electrode, the Co contents and the Co atomic weight, assuming each Co center accounts for one active site:

$$\# \text{ active sites } = \left(\frac{\text{catalyst loading per geometric area}(x\text{ g/cm}^2)\times\text{Co wt\%}}{\text{Co M}_\text{W}(\text{g/mol})}\right)\left(\frac{6.022\times10^{23}\text{ Co atoms}}{1\text{ mol Co}}\right) \qquad (3)$$

$$= \left(\frac{0.285\ \times10^{-3}\text{g/cm}^2\ \times\ 2.48\text{ wt\%}}{58.93\text{ g/mol}}\right)\left(\frac{6.022\ \times\ 10^{23}\text{ Co atoms}}{1\text{ mol Co}}\right)$$

$$= 7.2\ \times\ 10^{16}\text{ Co sites per cm}^2$$

Finally, the current density from the LSV polarization curve can be converted into TOF values according to:

$$\text{TOF} = \left(\frac{3.12\times10^{15}}{7.2\times10^{16}}\times\ |j|\right) = 0.0435\ \times\ |j| \qquad (4)$$

To determine the TOF value at thermodynamic potential (0 V *vs* RHE), with the $j = j_0 = 0.125$ mA cm^{-2}, where j_0 is the exchange current, it yields: TOF (0 V) = 0.0054 H_2 s^{-1}.

Supplementary Note 10: Electrochemically active surface area (ECSA) and active sites density measurements

The ECSA for the Co-NG electrode with mass loading of 285 μg cm^{-2} was estimated from the electrochemical double-layer capacitance (C_{dl}) of the catalytic surface.[28] The C_{dl} was determined

from the scan-rate dependence of CVs in a potential range where there is no Faradic current. The results were shown in Supplementary Fig. 19, which yields C_{dl} = 29.5 mF cm^{-2}. The ECSA can be calculated from the C_{dl} according to:

$$\mathrm{ECSA} = \frac{C_{\mathrm{dl}}}{C_{\mathrm{s}}} \quad (5)$$

where C_s is the specific capacitance of a flat standard electrode with 1 cm^2 of real surface area, which is generally in the range of 20 to 60 μF cm^{-2}.[17] If we use the averaged value of 40 μF cm^{-2} for the flat electrode, we obtain:

$$\mathrm{ECSA} = \frac{C_{\mathrm{dl}}}{C_{\mathrm{s}}} = \frac{29.5\ \mathrm{mF\ cm^{-2}}}{40\ \mathrm{\mu F\ cm^{-2}\ per\ cm^2_{ECSA}}} = 738\ \mathrm{cm^2_{ECSA}} \quad (6)$$

If we divide the as-obtained ECSA by the loading density of Co centers on the electrode (Co sites per cm^2), we can get the averaged area to find one Co center (cm^2 per site):

$$A_{\mathrm{ECSA\ per\ site}} = \frac{\mathrm{ECSA}}{\#\ \mathrm{active\ sites}} = \frac{738\ \mathrm{cm^2_{ECSA} per\ cm^2_{real}}}{7.2 \times 10^{16} \mathrm{Co\ sites\ per\ cm^2_{real}}} = 1.03 \times 10^{-14}\ \mathrm{cm}^2_{ECSA} \mathrm{per\ Co\ or\ } 1.03\ \mathrm{nm}^2_{ECSA} \mathrm{per\ Co} \quad (7)$$

This corresponds to ~20 benzene per Co in the Co-NG catalyst, assuming one benzene ring has an area of 0.05 nm^2.

The active sites density can be obtained by the inverse of the $A_{\mathrm{ECSA\ per\ site}}$:

$$\mathrm{Active\ sites\ denstiy\ (sites\ cm^{-2})} = \frac{1}{A_{\mathrm{ECSA\ per\ site}}} = 9.7 \times 10^{13}\ \mathrm{sites\ cm^{-2}} \quad (8)$$

Supplementary Note 11: Characterizations of the catalysts after cycling performance

The catalysts after cycling were firstly purified by at least five cycles of repeated centrifugation and redispersion in ethanol to get rid of the nafion, which was used as polymer binder during the preparation of electrodes. Then, the washed catalysts were dried and collected to allow further characterizations. The XPS survey spectrum is shown in Supplementary Fig. 20, which shows the presence of C, N and O, along with F and S resulting from the residue nafion and electrolyte, respectively. The Co 2p and N 1s spectra are shown in Supplementary Fig. 21. The peak positions and shapes are similar to those before cycling, indicating the chemical states of Co and N were not altered by cycling. The cycled sample were also characterized by XRD and compared to that before cycling. The results (Supplementary Fig. 22) show that in both samples there were peaks corresponding to the graphitic structure, but no peaks from Co-derived particles were observed. Finally, the cycled sample was characterized by STEM. The HAADF image (Supplementary Fig. 23) shows that the cobalt remains in atomic scale without severe aggregation. Efforts to get clearer images failed, probably due to the difficulty in the complete removal of the binder nafion. From the above analysis, it can be concluded that this Co-NG catalyst is stable in structure and electrochemical performance.

Supplementary Note 12: Calibration of reference electrodes

Hg/$HgSO_4$, K_2SO_4 (sat) and Hg/HgO, NaOH (1 M) reference electrodes were both calibrated with respect to the reversible hydrogen electrode (RHE). The calibration was conducted in a H_2-saturated electrolyte with Pt wires as both the working electrode and counter electrode. CVs were performed at a scan rate of 1 mV s^{-1}, and the averaged value of the two potentials at which

the anodic and cathodic scan crossed zero current was taken to be the thermodynamic potential for the hydrogen electrode reaction. According to the results shown in Supplementary Fig. 24 and Supplementary Fig. 25, in 0.5 M H_2SO_4, E (RHE) = E ($Hg/HgSO_4$) + 0.702 V, while in 1 M NaOH, E (RHE) = E (Hg/HgO) + 0.901 V.

Supplementary references

1. Zhou, J.; Duchesne, P. N.; Hu, Y.; Wang, J.; Zhang, P.; Li, Y.; Regier, T. & Dai, H. Fe–N bonding in a carbon nanotube–graphene complex for oxygen reduction: an XAS study. *Physical Chem. Chem. Phys.***16**, 15787-15791 (2014).
2. Kibsgaard, J.; Jaramillo, T. F. & Besenbacher, F. Building an appropriate active-site motif into a hydrogen-evolution catalyst with thiomolybdate $[Mo_3S_{13}]^{2-}$ clusters. *Nature Chem.* **6**, 248-253 (2014).
3. Li, Y.; Wang, H.; Xie, L.; Liang, Y.; Hong, G. & Dai, H. MoS_2 nanoparticles grown on graphene: an advanced catalyst for the hydrogen evolution reaction. *J. Am. Chem. Soc.* **133**, 7296-7299 (2011).
4. Xie, J.; Zhang, H.; Li, S.; Wang, R.; Sun, X.; Zhou, M.; Zhou, J.; Lou, X. W. & Xie, Y. Defect-rich MoS_2 ultrathin nanosheets with additional active edge sites for enhanced electrocatalytic hydrogen evolution. *Adv. Mater.* **25**, 5807-5813 (2013).
5. Xie, J.; Zhang, J.; Li, S.; Grote, F.; Zhang, X.; Zhang, H.; Wang, R.; Lei, Y.; Pan, B. & Xie, Y. Controllable disorder engineering in oxygen-incorporated MoS_2 ultrathin nanosheets for efficient hydrogen evolution. *J. Am. Chem. Soc.* **135**, 17881-17888 (2013).

6. Kibsgaard, J.; Chen, Z.; Reinecke, B. N. & Jaramillo, T. F. Engineering the surface structure of MoS_2 to preferentially expose active edge sites for electrocatalysis. *Nature Mater.* **11**, 963-969 (2012).
7. Li, D. J.; Maiti, U. N.; Lim, J.; Choi, D. S.; Lee, W. J.; Oh, Y.; Lee, G. Y. & Kim, S. O. Molybdenum sulfide/N-doped CNT forest hybrid catalysts for high-performance hydrogen evolution reaction. *Nano Lett.* **14**, 1228-1233 (2014).
8. Voiry, D.; Salehi, M.; Silva, R.; Fujita, T.; Chen, M.; Asefa, T.; Shenoy, V. B.; Eda, G. & Chhowalla, M. Conducting MoS_2 nanosheets as catalysts for hydrogen evolution reaction. *Nano Lett.* **13**, 6222-6227 (2013).
9. Merki, D.; Fierro, S.; Vrubel, H. & Hu, X. Amorphous molybdenum sulfide films as catalysts for electrochemical hydrogen production in water. *Chem. Sci.* **2**, 1262-1267 (2011).
10. Benck, J. D.; Chen, Z.; Kuritzky, L. Y.; Forman, A. J. & Jaramillo, T. F. Amorphous molybdenum sulfide catalysts for electrochemical hydrogen production: insights into the origin of their catalytic activity. *ACS Catal.* **2**, 1916-1923 (2012).
11. Cheng, L.; Huang, W.; Gong, Q.; Liu, C.; Liu, Z.; Li, Y. & Dai, H. Ultrathin WS_2 nanoflakes as a high-performance electrocatalyst for the hydrogen evolution reaction. *Angew. Chem. Int. Ed.* **53**, 7860-7863 (2014).
12. Yang, J.; Voiry, D.; Ahn, S. J.; Kang, D.; Kim, A. Y.; Chhowalla, M. & Shin, H. S. Two-dimensional hybrid nanosheets of tungsten disulfide and reduced graphene oxide as catalysts for enhanced hydrogen evolution. *Angew. Chem. Int. Ed.* **52**, 13751-13754 (2013).
13. Voiry, D.; Yamaguchi, H.; Li, J.; Silva, R.; Alves, D. C. B.; Fujita, T.; Chen, M.; Asefa, T.; Shenoy, V. B.; Eda, G. & Chhowalla, M. Enhanced catalytic activity in strained chemically exfoliated WS_2 nanosheets for hydrogen evolution. *Nature Mater.* **12**, 850-855 (2013).

14. Liu, Q.; Tian, J.; Cui, W.; Jiang, P.; Cheng, N.; Asiri, A. M. & Sun, X. Carbon nanotubes decorated with CoP nanocrystals: a highly active non-noble-metal nanohybrid electrocatalyst for hydrogen evolution. *Angew. Chem. Int. Ed.* **53**, 6710-6714 (2014).

15. Popczun, E. J.; Read, C. G.; Roske, C. W.; Lewis, N. S. & Schaak, R. E. Highly active electrocatalysis of the hydrogen evolution reaction by cobalt phosphide nanoparticles. *Angew. Chem.***126**, 5531-5534 (2014).

16. Xing, Z.; Liu, Q.; Asiri, A. M. & Sun, X. Closely interconnected network of molybdenum phosphide nanoparticles: a highly efficient electrocatalyst for generating hydrogen from water. *Adv. Mat.* **26**, 5702-5207 (2014).

17. Kibsgaard, J. & Jaramillo, T. F. Molybdenum phosphosulfide: an active, acid-stable, earth-abundant catalyst for the hydrogen evolution reaction. *Angew. Chem. Int. Ed.* **53**, 14433-14437 (2014).

18. Popczun, E. J.; McKone, J. R.; Read, C. G.; Biacchi, A. J.; Wiltrout, A. M.; Lewis, N. S. & Schaak, R. E. Nanostructured nickel phosphide as an electrocatalyst for the hydrogen evolution reaction. *J. Am. Chem. Soc.* **135**, 9267-9270 (2013).

19. Vrubel, H. & Hu, X. Molybdenum boride and carbide catalyze hydrogen evolution in both acidic and basic solutions. *Angew. Chem.* **124**, 12875-12878 (2012).

20. Zou, X.; Huang, X.; Goswami, A.; Silva, R.; Sathe, B. R.; Mikmeková, E. & Asefa, T. Cobalt-embedded nitrogen-rich carbon nanotubes efficiently catalyze hydrogen evolution reaction at all pH values. *Angew. Chem. Int. Ed.* **53**, 4372-4376 (2014).

21. Deng, J.; Ren, P.; Deng, D.; Yu, L.; Yang, F. & Bao, X. Highly active and durable non-precious-metal catalysts encapsulated in carbon nanotubes for hydrogen evolution reaction. *Energy Environ. Sci.* **7**, 1919-1923 (2014).

22. Zheng, Y.; Jiao, Y.; Li, L. H.; Xing, T.; Chen, Y.; Jaroniec, M. & Qiao, S. Z. Toward design of synergistically active carbon-based catalysts for electrocatalytic hydrogen evolution. *ACS Nano* **8**, 5290-5296 (2014).

23. Zheng, Y.; Jiao, Y.; Zhu, Y.; Li, L. H.; Han, Y.; Chen, Y.; Du, A.; Jaroniec, M. & Qiao, S. Z. Hydrogen evolution by a metal-free electrocatalyst. *Nature Commun.* **5**, 3783 (2014).

24. Zhao, Y.; Zhao, F.; Wang, X.; Xu, C.; Zhang, Z.; Shi, G. & Qu, L. Graphitic carbon nitride nanoribbons: graphene-assisted formation and synergic function for highly efficient hydrogen evolution. *Angew. Chem. Int. Ed.* **53**, 13934-13939 (2014).

25. Ito, Y.; Cong, W.; Fujita, T.; Tang, Z. & Chen, M. High catalytic activity of nitrogen and sulfur co-doped nanoporous graphene in the hydrogen evolution reaction. *Angew. Chem. Int. Ed.* **54**, 2131-2136 (2014).

26. Huang, X.; Zhao, Y.; Ao, Z. & Wang, G. Micelle-template synthesis of nitrogen-doped mesoporous graphene as an efficient metal-free electrocatalyst for hydrogen production. *Sci. Rep.* **4**, 7557 (2014).

27. Dupin, J. C.; Gonbeau, D.; Vinatier, P. & Levasseur, A. Systematic XPS studies of metal oxides, hydroxides and peroxides. *Phys. Chem. Chem. Phys.* **2**, 1319-1324 (2000).

28. McCrory, C. C. L.; Jung, S.; Peters, J. C. & Jaramillo, T. F. Benchmarking Heterogeneous Electrocatalysts for the Oxygen Evolution Reaction. *J. Am. Chem. Soc.* **135**, 16977-16987 (2013).
